# Inter-subunit energy transfer processes in a minimal plant photosystem II supercomplex

**DOI:** 10.1126/sciadv.adh0911

**Published:** 2024-02-23

**Authors:** Hoang Long Nguyen, Thanh Nhut Do, Kai Zhong, Parveen Akhtar, Thomas L. C. Jansen, Jasper Knoester, Stefano Caffarri, Petar Lambrev, Howe-Siang Tan

**Affiliations:** ^1^ School of Chemistry, Chemical Engineering and Biotechnology, Nanyang Technological University, 21 Nanyang Link, Singapore 637371.; ^2^ Zernike Institute for Advanced Materials, University of Groningen, Nijenborgh 4, 9747 AG Groningen, Netherlands.; ^3^ ELI-ALPS, ELI-HU Nonprofit Limited, Wolfgang Sandner utca 3, Szeged 6728, Hungary.; ^4^ HUN-REN Biological Research Centre, Szeged, Temesvári körút 62, Szeged 6726, Hungary.; ^5^ Faculty of Science, Leiden University, Einsteinweg 55, NL-2300 RA Leiden, Netherlands.; ^6^ Aix Marseille Université, CEA, CNRS, BIAM, LGBP, 13009 Marseille, France.

## Abstract

Photosystem II (PSII) is an integral part of the photosynthesis machinery, in which several light-harvesting complexes rely on inter-complex excitonic energy transfer (EET) processes to channel energy to the reaction center. In this paper, we report on a direct observation of the inter-complex EET in a minimal PSII supercomplex from plants, containing the trimeric light-harvesting complex II (LHCII), the monomeric light-harvesting complex CP26, and the monomeric PSII core complex. Using two-dimensional (2D) electronic spectroscopy, we measure an inter-complex EET timescale of 50 picoseconds for excitations from the LHCII-CP26 peripheral antenna to the PSII core. The 2D electronic spectra also reveal that the transfer timescale is nearly constant over the pump spectrum of 600 to 700 nanometers. Structure-based calculations reveal the contribution of each antenna complex to the measured inter-complex EET time. These results provide a step in elucidating the full inter-complex energy transfer network of the PSII machinery.

## Teaser

A direct measurement of ultrafast energy transfer from light-harvesting antennas to the photosystem II core is reported.

## INTRODUCTION

Photosynthesis provides plants and photosynthetic bacteria with the ability to use the widely available solar energy to produce storable chemical energy. In oxygenic photosynthesis, photosystem II (PSII) is responsible for the first steps of the whole photosynthetic process, which involves harvesting light energy to drive the photochemical water splitting reaction (*1*). The photochemical reactions of PSII produce oxygen as a by-product, essential for most organisms on Earth. Fully understanding the multistep complexity of the PSII photochemical process is, therefore, of great scientific interest. The lessons learned in natural photosynthesis can be applied to develop photoelectrochemical systems (*2*) and other artificial, advanced solar utilization technologies (*3*), important in powering the renewable energy economy for future global growth and sustainability.

PSII is a supercomplex constituted of various pigment-protein complexes that can be divided into two groups: the peripheral light-harvesting antennas and the core complex (CC). These pigment-protein complexes contain pigments such as carotenoids and chlorophylls (Chls) *a* and *b*, which absorb photons in the visible spectrum (*1*). The photoinduced biochemical processes in PSII are complex and comprise several steps. First, light harvesting is performed when photons are absorbed by pigments in pigment-protein complexes, dominated by the peripheral trimeric light-harvesting complex II (LHCII). This is followed by excitation energy transfer (EET) processes that eventually transfer the energy to the reaction centers (RCs) in the CC to perform the primary charge separation, which subsequently yields water splitting (*1*). The EET processes in PSII can broadly be divided into intra-complex and inter-complex EET and can occur at timescales from femtoseconds to hundreds of picoseconds (*4**–**6*).

Through the years, much effort has been expended to clarify and understand these various processes. Recently, the structure of the PSII has been solved at 2.7-Å resolution (*7**–**9*), providing scientists a closer look into how the exact arrangements of the pigment molecules and the protein structure govern the EET processes (*6*). Intra-complex EET, especially within LHCII, has been well studied (*10**–**17*). Generally, in LHCII, EET from carotenoids and Chls *b* to Chls *a* happens at sub-picosecond timescales, while energy equilibration between Chls *a* proceeds up to several picoseconds (*6*, *10*, *12*, *14*, *17**–**19*). The dynamics of other monomeric PSII antenna complexes (CP24, CP26, and CP29) are similar to that of LHCII, due to their homology in structures (*20*), although they have received less attention. The minor antennas are mostly responsible for specific roles, such as photoprotection and creating an energetic and structural bridge between the larger complexes (*20**–**25*). Recently, a study was carried out on the assembly of LHCII(M) (moderately bound LHCII in the PSII supercomplex), CP29, and CP24 (*25*). The EET dynamics were shown to be different between LHCII in the assembly and in isolated form.

The EET within the PSII CC has also been much studied. The PSII CC consists of three main functional units: two internal antennas (CP43 and CP47) and the RC (consisting of the D1 and D2 proteins, cytochrome *b*_559_, the oxygen-evolving complex, and several smaller subunits). The structure of the PSII CC is highly conserved across different organisms (*26*). CP43 and CP47 are responsible for both light-harvesting and funneling energy flows from the peripheral antennas (LHCII and the monomeric antenna complexes) to the RC. The timescales of EET from CP43/CP47 to the RC are still being debated (*6*, *27*). Specifically, a dominating ~40-ps lifetime in the PSII CC appears in various time-resolved fluorescence studies (*27**–**30*). This time constant results from both the migration of energy from CP43/CP47 to the RC and the charge separation process in the RC. In the case that the energy transfer to the RC is much slower than the charge separation process, it is termed the transfer–to–trap-limited case (*31**–**33*), while the opposite case is termed trap-limited (*28*, *30*). As the size of the peripheral antenna attached to the CC increases, the kinetic limitation increasingly turns into the diffusion length of the excitations through the antenna and energy migration to the CC, which, in turn, depends on the inter-complex rates of EET.

A major keystone necessary for the understanding of the function of PSII is the elucidation of the inter-complex EET processes underlining the flow of energy collected by peripheral antenna complexes to the PSII CC (*1*). However, to date, there has not been any ultrafast spectroscopic study that directly measures such inter-complex EET processes in PSII. Hitherto, experimental studies of the inter-complex EET lifetimes in PSII have been scarce and only provided indirect estimations. Previous studies used time-resolved fluorescence of PSII supercomplexes and data analysis with coarse-grained kinetic modeling, wherein each individual complex is treated as a “supersite” (*5*, *34*, *35*). By fitting the modeled kinetics to the fluorescence data, the effective exciton hopping times between complexes have been estimated (*5*, *6*). Theoretical simulations of the dynamics of the PSII supercomplex have also been performed with the use of structure-based calculations. Several approaches have been reported to describe exciton dynamics, using approximate methods like modified Redfield–generalized Förster rate theories (*36*, *37*), diffusion in fluctuating systems (*38*), as well as the more accurate hierarchical equations of motion (HEOMs) (*39*). These studies, however, only treated the inter-complex EET in a diffusion picture and focused on the efficiency of the PSII supercomplex in the thylakoid membrane for trapping excitation energy. Another study using a non-Markovian quantum master equation with zeroth-order functional expansion (*40*) provided some details about the antenna-to-core EET, in the LHCII-CP43-RC system.

Two-dimensional electronic spectroscopy (2DES) can be considered an advanced form of ultrafast transient absorption (TA) spectroscopy. On a resulting 2D spectrum, correlating excitation wavelengths to detection wavelengths, one can visualize any excitation wavelength–dependent dynamics in a single consistent set of measurements (*41*). 2DES has been used to elucidate the inter-complex EET in other photosynthetic systems, such as the green sulfur bacteria (*42*) and phycobilisomes (*43*, *44*). However hitherto, no ultrafast TA or 2DES studies have been performed to elucidate the inter-complex EET in PSII supercomplexes. 2DES has proved to be useful in studying the EET dynamics of component complexes in plant PSII (*10*, *17*, *18*, *22*, *45**–**50*). In this study, we apply 2DES to directly measure the inter-complex EET between the peripheral antenna complexes and the PSII CC in a small plant PSII supercomplex. We study three systems at 80 K: (i) the smallest purifiable PSII supercomplex containing the monomeric CC and the peripheral antennas [LHCII(S) and CP26], henceforth indicated as the C_1_S_1_ complex (*51*); (ii) the isolated peripheral antenna system (LHCII trimer); and (iii) the isolated monomeric PSII CC. The differences in the dynamics of the 2D electronic spectral features between C_1_S_1_, isolated LHCII, and the CC can then be extracted to reveal the inter-complex EET between the components. We observe and determine this energy transfer process to have an effective timescale of 50 ps. The EET time is also independent of the excitation wavelength. We further perform structure-based calculations using Redfield–generalized Förster theories. By comparing the calculations to our experimentally measured values, we obtain additional details about the contributions of each antenna complex to the observed energy transfer timescale to the CC in the PSII supercomplex.

## RESULTS

### Spectroscopic features of the C_1_S_1_ complex

**Fig. 1.**
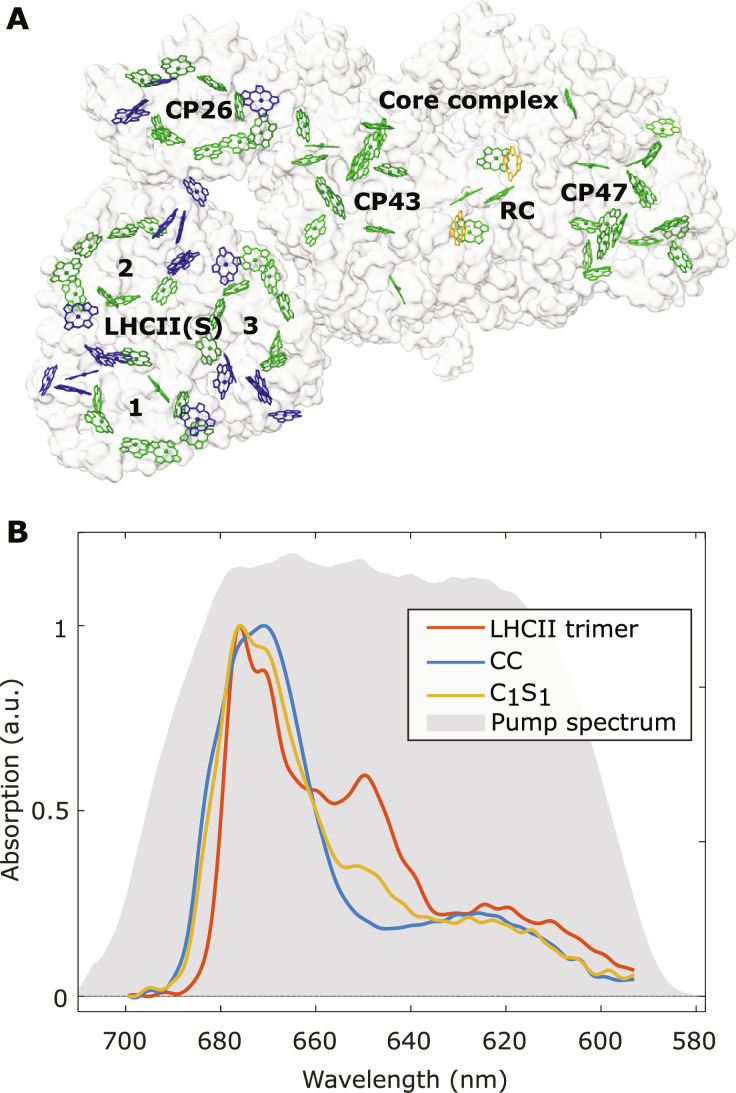
The structure and linear absorption spectrum of the C_1_S_1_ complex. (**A**) The arrangement of Chl *a* (green), Chl *b* (blue), and pheophytin (yellow) pigments in the C_1_S_1_ complex (Protein Data Bank code 5XNM). The apoprotein surface is rendered in white. The names of the component pigment-protein complexes are denoted. LHCII(S) monomers are also labeled with numbers. (**B**) Linear absorption spectra at 80 K of the LHCII trimer (red), CC (blue), and C_1_S_1_ complex (yellow), normalized to the *Q_y_* maxima. a.u., arbitrary units.

Figure 1A shows the Chl arrangement in the C_1_S_1_ complex resolved from the cryo–electron microscopy (cryo-EM) structure (*7*), looking from the stromal side of the thylakoid membrane. Chls *a* and *b* are both found in the LHCII(S) trimer (24 Chls *a* and 18 Chls *b*) and CP26 (9 Chls *a* and 4 Chls *b*), while the CC contains 35 Chls *a* (13 in CP43, 16 in CP47, and 6 in the RC) and two pheophytin *a* molecules. The contrast in pigment compositions between the complexes can be observed in their linear absorption spectra at 80 K (Fig. 1B). Here, the spectra are normalized to the maxima of the Chl *a Q_y_* band, and the Chl *b*/*a* ratio in each complex is indicated by the relative absorbance at ~650 nm, which is highest in LHCII (*b*/*a* ratio of 0.75), followed by C_1_S_1_ (0.32) and eventually CC (0).

In Fig. 1B, it can be observed that the main absorption region in LHCII (660 to 680 nm) features two sharp peaks at around 670 and 678 nm. On the other hand, the absorption spectrum of the CC is much broader and slightly extended to longer wavelengths (>680 nm), reflecting the lower-energy pools present in the CC (*48*, *52*). This is due to the dense concentration of Chls *a* and the distinct protein environment in the CC (*26*, *48*, *53**–**55*). The absorption spectrum of the C_1_S_1_ complex ultimately contains the features of both LHCII and the CC.

**Fig. 2.**
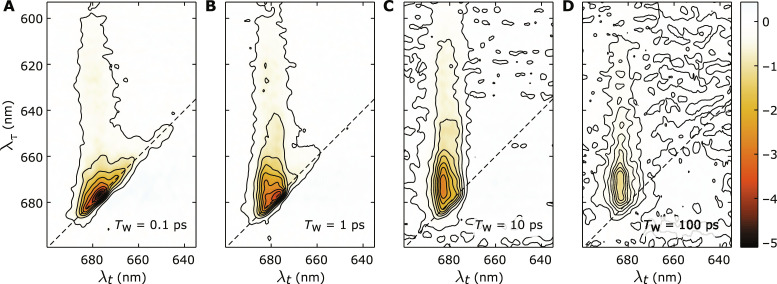
**Absorptive 2D spectra of the C_1_S_1_ complex at various waiting times, *T*_w_. *T*_w_ = (**A**) 0.1, (**B**) 1,(**C**)10, and(**D**) 100 ps. The color scales among the spectra are identical and shown on the right end. The vertical axes represent the excitation wavelengths, λ_τ_, while the horizontal axes represent the detection wavelengths, λ_*t*_.

Representative 2D spectra of the C_1_S_1_ complex at selected waiting times *T*_w_ are shown in Fig. 2. The 2D spectra of LHCII and the CC can be found in the Supplementary Materials. In the 2D spectra, the vertical and horizontal axes represent excitation and detection wavelengths, λ_τ_ and λ*_t_*, respectively. The pump spectrum in our experiment covers the wavelength range of 590 to 700 nm (Fig. 1B). The broad spectrum allows us to almost evenly excite all *Q* band transitions of Chls, including the *Q_y_* manifold of Chls *a* and *b* and part of the higher-energy transitions (*Q_x_* and vibronic progressions).

Generally, the detected 2D signals migrate along the λ*_t_* axis from short to long wavelengths with waiting time *T*_w_, depicting the EET from high- to low-energy states in the protein complexes. At each excitation wavelength λ_τ_, the energy flow exhibits different dynamics depending on the excited state. Excitations in the *Q_x_* and vibronic region (λ_τ_ < 640 nm) relax very rapidly to the low-energy states, at sub–100-fs timescales. This can be observed in Fig. 2A, where at λ_τ_ < 640 nm, no diagonal peaks are observed and the cross peaks are mostly detected at λ*_t_* ~ 680 nm. At excitation wavelengths λ_τ_ = 645 to 655 nm, we observe a relaxation of the diagonal peak within 1 ps that results in the formation of cross peaks at λ*_t_* ~ 675 nm (Fig. 2B). This corresponds to Chl *b*→*a* EET processes as observed more prominently in the spectra of LHCII (see fig. S1). At excitation wavelengths λ_τ_ = 660 to 670 nm, the kinetics involve the relaxation of intermediate Chl *a* states, which mainly happens after 1 ps and is indicated by the disappearance of diagonal peaks at around 10 ps (Fig. 2C). The lowest excitation region at λ_τ_ = 670 to 690 nm shows the equilibration between the Chl *a* pools (*6*, *12*, *14*, *17*, *49*). Beyond *T*_w_ = 100 ps, most of the EET processes are completed and the intensities of the 2D signals decay due to population relaxation (Fig. 2D).

The 2D electronic spectra of C_1_S_1_ contain spectral contributions from its components, i.e., LHCII, CP26, and the CC. For example, the diagonal signal at 650 nm appearing at *T*_w_ = 0.1 ps (Fig. 2A) originates from Chls *b* that only exist in LHCII and CP26 (see fig. S1). The strong diagonal peak at around 678 nm at early *T*_w_’s (Fig. 2, A and B) is also typical for LHCII. The diagonal signals in C_1_S_1_ also extend to longer wavelengths, up to around 684 nm, which, on the other hand, is a feature of the 2D diagonal signals of the CC (see fig. S2). From *T*_w_ = 1 ps onward, most cross peaks can be detected at two distinct wavelengths, λ*_t_* = 678 and 684 nm, and can largely be assigned to be the equilibrated populations within LHCII-CP26 and CC, respectively.

In addition, the ratio between cross peak signals detected at λ*_t_* = 678 and 684 nm at early *T*_w_’s is largely correlated with the linear absorption ratio between the peripheral antennas and the CC shown in Fig. 1B. In particular, at excitation wavelengths λ_τ_ = 645 to 655 nm, the cross peaks are due to the relaxation of the excited state population in Chl *b* in LHCII-CP26; thus, the detected signals are more concentrated at λ*_t_* = 678 nm of LHCII-CP26 (Fig. 2B). In contrast, at λ_τ_ = 660 to 680 nm, more cross peak signals are observed at the lower-energy state (λ*_t_* = 684 nm; Fig. 2B), because the CC features a broader absorption in this region. Notably, at long *T*_w_’s (Fig. 2, C and D), we detect signals mostly at λ*_t_* = 684 nm at all excitation wavelengths.

**Fig. 3.**
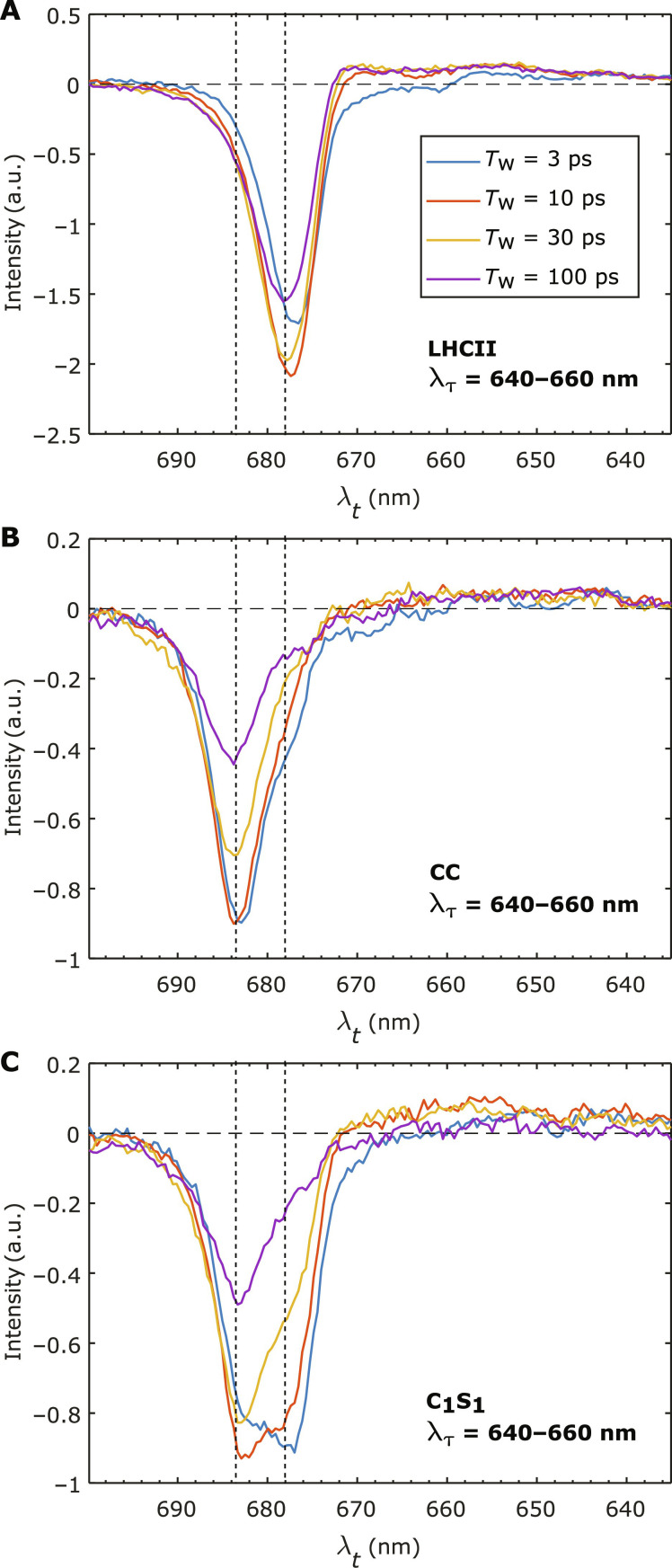
Integrated 2D spectra over the Chl *b* absorption region (640 to 660 nm). The integrated 2D spectra of (**A**) LHCII, (**B**) CC, and (**C**) C_1_S_1_ are shown at various waiting times *T*_w_ from 3 to 100 ps. Two dashed lines highlight the detection wavelengths of λ*_t_* = 678 and 684 nm to indicate the positions of the fingerprint bleach signals of LHCII and the CC, respectively.

We now take a closer look at the 2D spectra of LHCII and the CC in comparison to those of C_1_S_1_. In Fig. 3 (A and B), we present the 2D spectra of LHCII and the CC, respectively, summed over the excitation wavelengths λ_τ_ = 640 to 660 nm. This corresponds to TA spectra excited at around the *Q_y_* absorption band of Chl *b*. The spectral region emphasizes the contributions from the peripheral antenna complexes (LHCII and CP26) as the CC does not contain Chl *b*. We shall henceforth term these summed 2D spectral signals as TA signals. From Fig. 3 (A and B), the TA signals show that transiting from *T*_w_ = 3 to 10 ps, there is a small change in the main bleach peaks of the TA spectra. However, the peaks then stay largely at the same position beyond 10 ps and center at λ*_t_* = 678 nm in LHCII and 684 nm in the CC. Beyond 10 ps, the TA signal of LHCII hardly changes, apart from a slight Stokes shift of ~1 nm and a decrease in amplitude (Fig. 3A). This is well attested to as the intra-complex EET dynamics in LHCII is effectively finished by ~10 ps (*10*, *56*). The decrease in amplitude after 10 ps is mostly due to spontaneous relaxation and exciton-exciton annihilation processes. We note that the excitation conditions in our experiments were low enough to minimize the effects of exciton-exciton annihilation. As shown in the Supplementary Materials (fig. S3), the TA of CP26 can be considered to be similar to those of LHCII (*20*). The equilibrated bleach signal of CP26 also centers at λ*_t_* = 678 nm, similar to LHCII. Hence, the negative bleach at 678 nm can be considered as a spectroscopic fingerprint of the excited antenna population (LHCII and CP26).

On the other hand, the CC TA signals exhibit a faster decay (Fig. 3B) as excitations are transferred from CP43/CP47 to the RC and trapped (*28*, *31*). Crucially, the main spectral feature with the bleach peak at λ*_t_* = 684 nm remains dominant over this timescale, which is also observed in earlier EET studies of the PSII CC (*48*). Although a charge separation spectral feature is also observed between 10 and 100 ps at λ*_t_* ~ 665 to 675 nm due to the electrochromic shift (*57*), it has a relatively minor contribution to the spectrum. Therefore, the 684-nm bleach feature can be used as the fingerprint to track the excited population of the CC.

Figure 3C shows the TA spectra of C_1_S_1_ over the same *T*_w_’s and excitation region. The TA spectra in C_1_S_1_ exhibit notable spectral evolutions extending from 3 to 100 ps. Beside the overall intensity decay of the whole spectrum with *T*_w_, the decay of the TA signal at λ_t_ = 678 nm is clearly correlated with the rise of the signal amplitude at 684 nm. The peak initially near 678 nm at *T*_w_ = 10 ps gradually evolves to a peak at near 684 nm by *T*_w_ = 100 ps. These two evolving spectral features at 678 and 684 nm coincide with the spectroscopic fingerprints of LHCII and the CC, as described above.

**Fig. 4.**
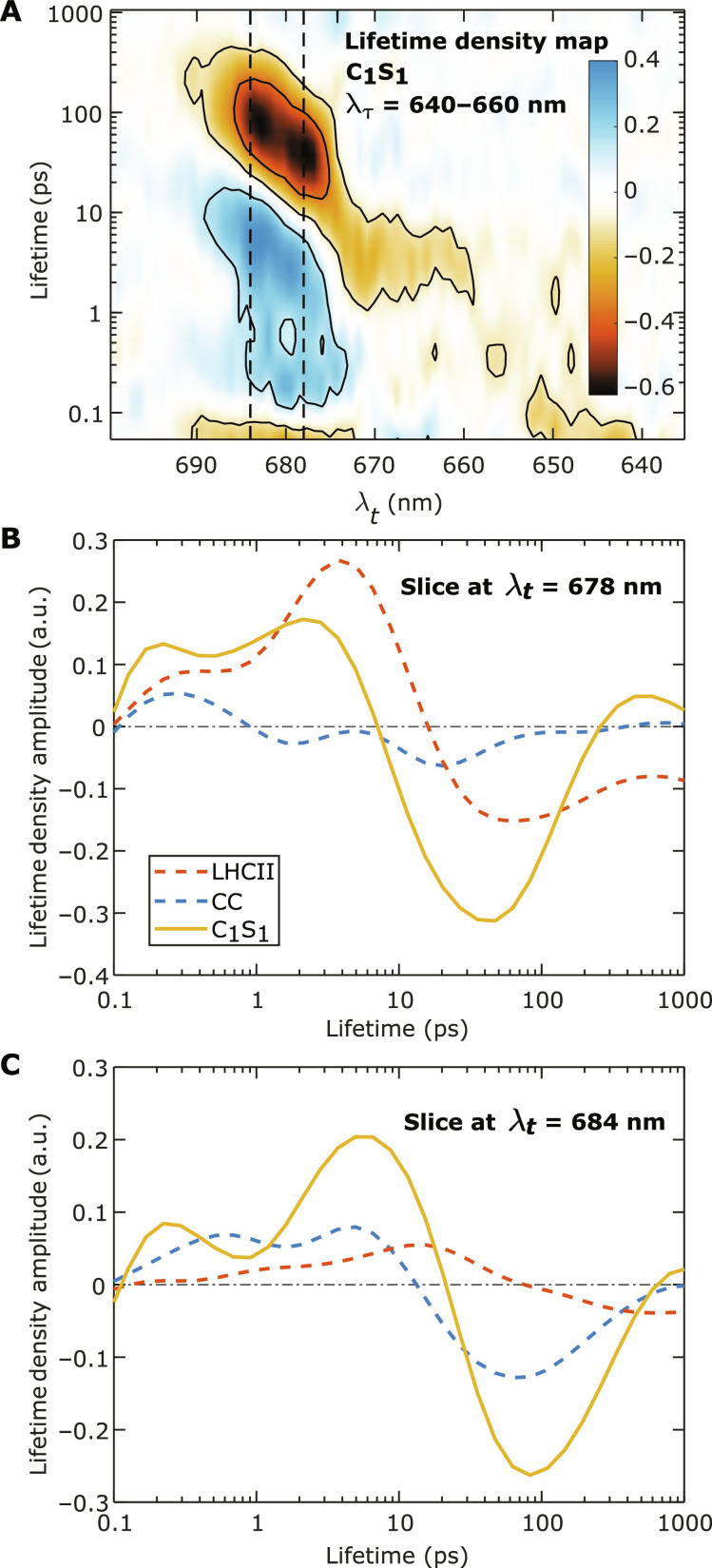
Lifetime densities of the 2D spectra of the C_1_S_1_ complex with the excited region at λ_τ_ = 640 to 660 nm. (**A**) The lifetime density map (LDM) of the integrated 2D spectra of the C_1_S_1_ complex. Negative/positive values represent decay/rise of the bleach signals. The LDM is obtained from LDA on the 2D spectra at *T*_w_ = 50 fs to 1.5 ns, with the regularization parameter α_LDA_^2^ = 0.1. Two dashed lines highlight the detection wavelengths of λ*_t_* = 678 and 684 nm to indicate the positions of the fingerprint bleach signals of LHCII and the CC, respectively. The lifetime density amplitudes along these two lines are shown in (**B**) and (**C**), respectively, in comparison with the lifetime densities from LHCII (dashed red) and the CC (dashed blue). The lifetime densities of LHCII and the CC are scaled to reflect the contributions of these two complexes in C_1_S_1_.

The timescale of the observed dynamics described above in the C_1_S_1_ complex can be further analyzed using lifetime density analysis (LDA) on the 2D spectroscopic data. We use the LDA procedure with Tikhonov regularization described in (*58*) and our previous work (*25*). Figure 4A shows an integrated lifetime density map (LDM) of the C_1_S_1_ 2D spectra with the excited region at λ_τ_ = 640 to 660 nm. The same LDMs of LHCII and the CC data are provided in the Supplementary Materials (fig. S4). As can be seen in Fig. 4A, at sub–10-ps lifetimes, the EET dynamics of C_1_S_1_ consists of various energy relaxation processes from high-energy states at λ*_t_* = 660 to 675 nm to low-energy states at λ*_t_* = 678 to 685 nm, consistent with the individual dynamics found in LHCII and the CC. Beyond 10 ps, decays to ground states can be observed at λ*_t_* = 678- and 684-nm positions, where the former decays at ~30 ps and the latter decays at ~100 ps.

In Fig. 4 (B and C), we show the lifetime density amplitudes along the λ*_t_* = 678- and 684-nm detection wavelengths (the slices along the two dashed lines in Fig. 4A) and compare them with those from LHCII and the CC. The lifetime densities for LHCII and the CC are scaled by 45 and 65%, respectively, of their original intensities to reflect their relative signal contributions in the C_1_S_1_ complex. The origins of the scaling factors are explained in the next section. In Fig. 4B, which depicts the lifetime density amplitudes at detection wavelength λ*_t_* = 678 nm, LHCII exhibits various decay processes with lifetimes from ~100 ps to several nanoseconds. This is consistent with the bleach peak stabilizing at ~678 nm and gradually decaying, as shown in Fig. 3A. The lifetime density for the CC does not show any notable amplitude. Meanwhile, in the C_1_S_1_ complex, strong decay components appear with a peak at the lifetimes from ~10 to 100 ps, peaking at ~30 to 40 ps. This is markedly different from the kinetics in LHCII, suggesting an extra process occurring at lifetimes of tens of picoseconds in the C_1_S_1_ complex. Crucially, the slower decay present in LHCII has disappeared in the C_1_S_1_ complex. In Fig. 4C, we show the lifetime density amplitudes detected at λ*_t_* = 684 nm. For the CC, there is a positive contribution at short lifetimes, up to ~10 ps, followed by a negative peak that peaks at ~60 ps. The former feature is consistent with the intra-complex EET dynamics within the CC, while the latter is consistent with the decay to a charge separation state (*57*). In comparison, the lifetime density for C_1_S_1_ bears a similar form as that of the CC but has the features shifted to longer lifetimes. In particular, the positive feature extends to ~20 ps, and the negative peak is shifted to ~80 ps.

We can now draw the hypothesis that the additional decay of the signal at 678 nm is correlated with a relative rise of signal at 684 nm as shown in Fig. 3C, and it is due to EET from the former to the latter. The hypothesis is also consistent with the comparisons of the lifetime density amplitudes at λ*_t_* = 678 and 684 nm between the LHCII, CC, and C_1_S_1_ complex in Fig. 4 (B and C). The observed spectral dynamics in C_1_S_1_ occurring from *T*_w_ = 10 to 100 ps is, thus, due to the inter-complex EET between the antennas (LHCII-CP26) and the CC. For completeness, we also consider another plausible possibility that the spectral contribution of the LHCII-CP26 (678-nm signals) decays faster than that of the CC (684-nm signals) with the intra-complex manner. This hypothesis, however, can be ruled out with the observation from Fig. 3 (A and B) that the CC signal decays at a faster rate than that of LHCII. The features from the TA spectra in Fig. 3C and the LDMs in Fig. 4 only provide a qualitative picture about the extra processes appearing in the C_1_S_1_ complex. To extract the inter-complex, antenna-to-core EET kinetics, we need to construct a kinetic model based on the hypothesis drawn above.

### Extracting the inter-complex EET dynamics

We construct a model as follows. We first denote the measured TA spectra of the isolated LHCII, isolated CC, and C_1_S_1_ (Fig. 3, A, B, and C, respectively) as S_LHCII_(λ*_t_*, *T*_w_), S_CC_(λ*_t_*, *T*_w_), and S_C1S1_(λ*_t_*, *T*_w_), respectively. These spectra include the detection range λ*_t_* from 640 to 700 nm. We assume that S_C1S1_(λ*_t_*, *T*_w_) at each *T*_w_ comprises a spectral component contributed by the LHCII-CP26 domain, and a spectral component contributed by the CC domain in C_1_S_1_. The former is assumed to be represented by S_LHCII_(λ*_t_*, *T*_w_) multiplied with a *T*_w_-dependent scaling factor, α(*T*_w_). Here, we have made the reasonable assumption that CP26 behaves similarly to LHCII, as mentioned earlier. Likewise, we assume that the spectral component by the CC domain can be represented by S_CC_(λ*_t_*, *T*_w_) multiplied with a *T*_w_-dependent factor β(*T*_w_). Therefore, at each *T*_w_, we can write

$$S_{C1S1}\left(T_{w}\right)=\alpha \left(T_{w}\right)S_{\text{LHCII}}\left(T_{w}\right)+\beta \left(T_{w}\right)S_{\text{CC}}\left(T_{w}\right)$$ (1)

**Fig. 5.**
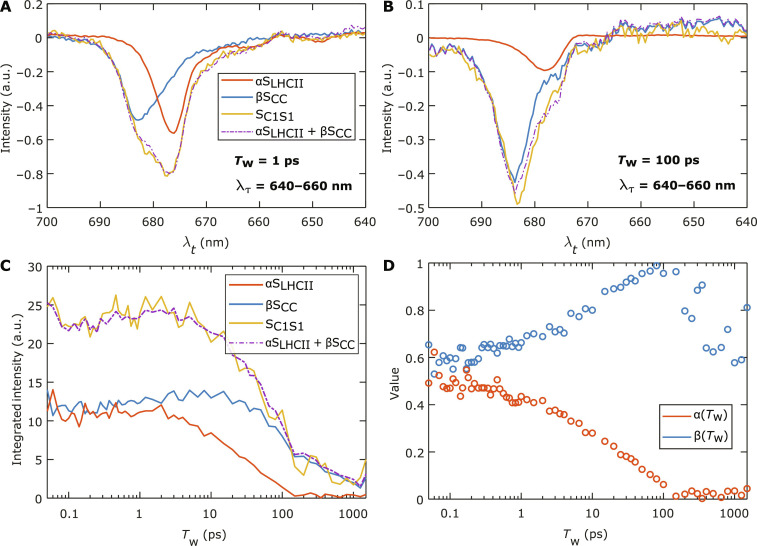
Results of the fits of C_1_S_1_ spectra with spectra from LHCII and the CC. The TA spectra of LHCII (red) and CC (blue), multiplied with their *T*_w_-dependent scaling factors (α and β) so that the summed TA spectrum (dash-dotted purple) fits the TA spectrum of the C_1_S_1_ complex (yellow). The TA spectra at *T*_w_ = 1 and 100 ps are shown in (**A**) and (**B**), respectively. The spectra are excited at the Chl *b* absorption region (640 to 660 nm). (**C**) The kinetic traces obtained by integrating the TA spectra along the λ*_t_* axis, at waiting times *T*_w_ from 50 fs to 1.5 ns, representing the excited state population evolution of C_1_S_1_ and its components (LHCII and the CC). (**D**) *T*_w_-dependent values of the scaling factors α and β.

The factors α(*T*_w_) and β(*T*_w_) are obtained at each waiting time *T*_w_ by a least-square fit to S_C1S1_(λ*_t_*, *T*_w_) with a linear combination of the measured S_LHCII_(λ*_t_*, *T*_w_) and S_CC_(λ*_t_*, *T*_w_). We note that both S_LHCII_(λ*_t_*, *T*_w_) and S_CC_(λ*_t_*, *T*_w_) spectra continue to evolve with *T*_w_. By using them at each *T*_w_ as the basis for the fit to S_C1S1_(λ*_t_*, *T*_w_), we can assume that the dynamics of α(*T*_w_) and β(*T*_w_) are directly tracking the dynamics of the antenna-to-core EET process. This can also be shown using a kinetic model for antenna-to-core EET (shown in the Supplementary Materials). One can notice that inter-complex processes may also result in a change in shape of the component spectra. This means that, by using S_LHCII_(λ*_t_*, *T*_w_) and S_CC_(λ*_t_*, *T*_w_) as the basis, it is assumed that the inter-complex processes are independent of the intra-complex processes. Examples of the results of the fit are shown in Fig. 5 (A and B), in which the TA spectra of C_1_S_1_ (S_C1S1_) excited at λ_τ_ = 640 to 660 nm are well described as the sum (dash-dotted purple) of the scaled spectra from LHCII (αS_LHCII_) and the CC (βS_CC_) at the respective *T*_w_. The fit quality is reflected by the small residues compared to the signal amplitudes (see fig. S5) and thereby adds confidence to the validity of our assumptions.

In Fig. 5C, we present the *T*_w_-dependent kinetic traces of C_1_S_1_ (yellow), LHCII (red), and CC (blue) signals. The traces are obtained by integrating the TA spectra αS_LHCII_, βS_CC_, and S_C1S1_ over the detection wavelength λ*_t_*. The summed kinetics of αS_LHCII_ and βS_CC_ is represented by the dash-dotted purple line, which follows closely the kinetics of S_C1S1_ and reflects the fit quality across the entire *T*_w_ range. By *T*_w_ = 100 ps, the kinetics of the integrated αS_LHCII_ has decayed almost completely, while S_C1S1_ contains only contributions from βS_CC_.

In Fig. 5D, the resultant α(*T*_w_) and β(*T*_w_) are plotted. At early *T*_w_, the two factors are nearly constant, as the TA spectra of C_1_S_1_ are expected to comprise a constant ratio between the contributions of LHCII and the CC because no appreciable inter-complex EET dynamics are happening on this timescale. The values at early *T*_w_ (~0.45 for α and ~0.65 for β) are used to scale the respective lifetime contributions in Fig. 4. Figure 5D shows that α(*T*_w_) exhibits a decay process to zero on the timescale of tens of picoseconds. This decay process is associated with a rise in β(*T*_w_). These observations indicate an energy transfer from LHCII-CP26 to the CC, and, from α(*T*_w_), we can determine the inter-complex EET rate. We also note that beyond *T*_w_ = 100 ps, β(*T*_w_) trends toward a decay. This is likely due to a higher exciton-exciton annihilation rate occurring with the large unit size of C_1_S_1_. We note that the experimental conditions, namely, the pump pulse fluences and the sample OD, used for all experiments in the three sets of samples were similar. Therefore, the number of photons absorbed is similar for all three types of samples. However, the C_1_S_1_ sample, with only about half of total particles compared to the LHCII and CC samples, will have higher excitation density per particle. This leads to a comparatively higher contribution of the exciton-exciton annihilation processes to the S_C1S1_(λ*_t_*, *T*_w_) spectrum compared to the S_CC_(λ*_t_*, *T*_w_) spectrum, and the fit, therefore, lowers the value of β(*T*_w_) to account for this discrepancy. However, because this effect only becomes visible beyond *T*_w_ = 100 ps, it will not have any substantial effect on our analysis of tens of picosecond processes.

**Fig. 6.**
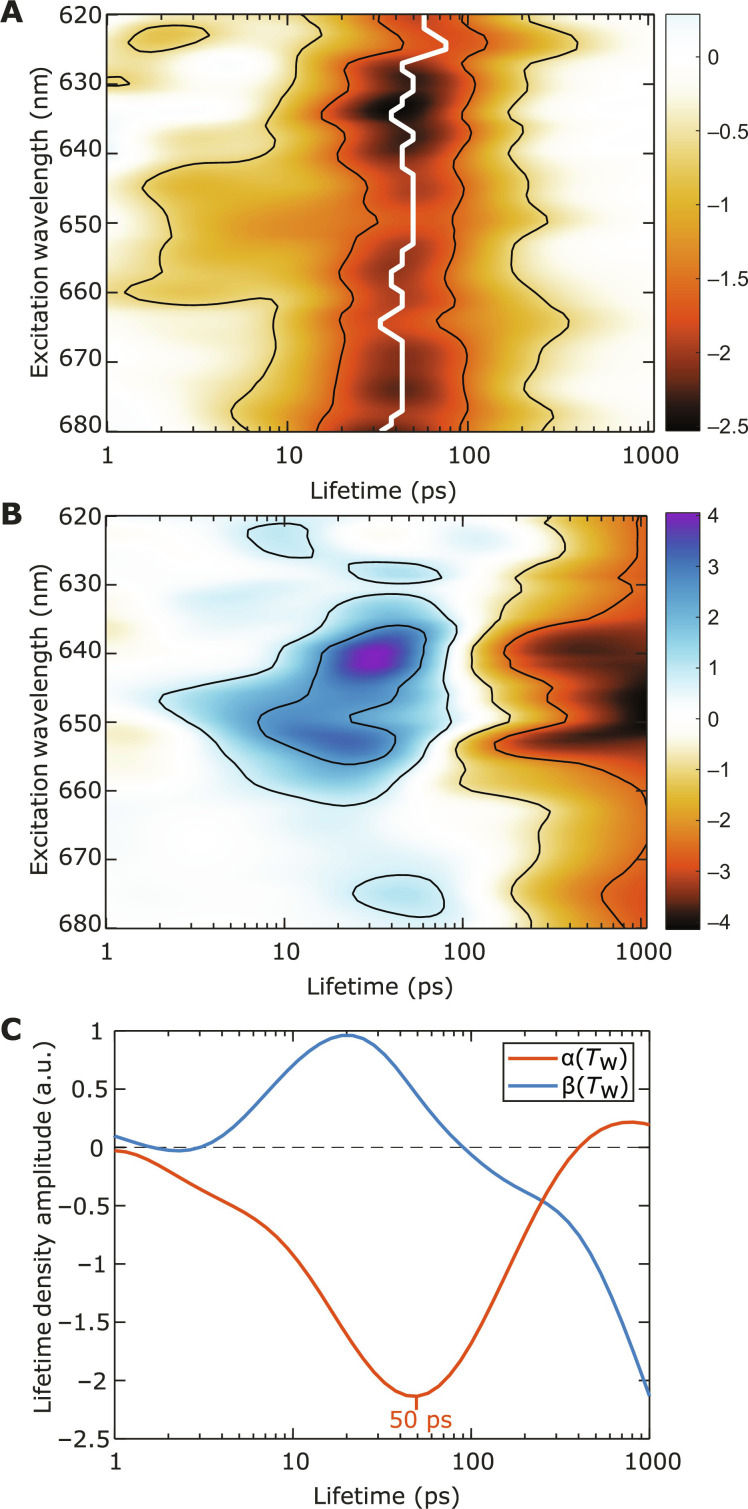
Lifetime densities of the scaling factors α(*T*_w_) and β(*T*_w_) at various excitation wavelength ranges. The LDMs of (**A**) α(*T*_w_) and (**B**) β(*T*_w_) obtained from the spectral analysis integrated at every excitation wavelength from 620 to 680 nm, with 10-nm bandwidths. The LDA is performed on data at *T*_w_ = 1 to 300 ps, with the regularization parameter α_LDA_^2^ = 0.5. The white line in (A) connects the minima at each excitation wavelength. (**C**) The lifetime density amplitudes of α(*T*_w_) (red) and β(*T*_w_) (blue) obtained from the spectral analysis integrated from λ_τ_ = 600 to 700 nm, showing the averaged inter-complex EET lifetimes across the pump spectrum. The LDA is performed on data at *T*_w_ = 50 fs to 1.5 ns, with the regularization parameter α_LDA_^2^ = 0.5.

In photosynthetic light-harvesting complexes, there exist multiple EET pathways connecting the complexes to the RC to maintain the overall robustness of the energy trapping process (*6*). Because each excitonic energy level is localized in certain domains of pigments, one may be able to achieve more specificity in the EET measurement from different starting domains by resolving the excitation wavelengths. 2DES provides the ability to resolve the excitation energy axis, thus allowing the analysis above to be applied consistently across all excitation wavelengths. We perform the analysis to the TA obtained from integrating the 2D spectra over a 10-nm excitation bandwidth at every excitation wavelength from 620 to 680 nm. Figure 6A shows the LDM of α(*T*_w_) obtained from such wavelength-dependent analysis. In the figure, there is a negative band at ~50 ps with the exact minima of the band highlighted by a white line. We can interpret these maxima, representing the major timescale of the decay of α(*T*_w_) as the inverse of the inter-complex EET rate. One important observation here is that this rate does not exhibit any major variation with excitation wavelengths. In Fig. 6B, the LDM of β(*T*_w_) is presented. Here, there is a positive band at lifetimes of ~40 to 50 ps, representing a rise in signal. Similar to the case of α(*T*_w_), the lifetime only exhibits little dependence on the excitation wavelengths. We note that, in Fig. 6B, compared to other excitation regions, the amplitudes in the Chl *b* absorption region (λ_τ_ = 640 to 660 nm) are higher. This reflects a stronger rise of β(*T*_w_) in this excitation region, which is due to more excitation created in the antenna instead of the CC (the CC does not contain Chls *b*). As mentioned earlier, the similar timescale of the decay in α(*T*_w_) and rise in β(*T*_w_) is consistent with the observation of an inter-complex EET process. The β(*T*_w_) LDM having maxima slightly earlier than that of the α(*T*_w_) LDM can be attributed to the effect of the subsequent >100-ps decay “pushing” the positive peak to an earlier time. We also note that the independence of the inter-complex EET lifetime on the excitation wavelength only applies to the observable process in our measurement, i.e., inter-complex processes with lifetimes slower than 10 ps.

It is also meaningful to consider the EET rate with an excitation over a broad section of the action spectrum for photosynthesis. We now perform analysis to the entire excitation window of λ_τ_ = 600 to 700 nm, covering the *Q_y_* transitions as well as a substantial part of *Q_x_* and vibronic bands of all Chl pigments. From the 2D spectra of LHCII, CC, and C_1_S_1_, similar analysis is carried out as in the previous section (Fig. 5) to recover α(*T*_w_) and β(*T*_w_) for an excitation window of λ_τ_ = 600 to 700 nm. The detailed results are shown in fig. S6. In Fig. 6C, we portray the lifetime density amplitudes of α(*T*_w_) and β(*T*_w_) obtained from this analysis. The lifetime density of α(*T*_w_) (red) peaks at 50 ps but has contributions ranging from tens of picoseconds to ~100 ps. The bandwidth of the lifetime density can vary with the degree of regularization used in the LDA, but the peak remains at ~50 ps. We can cite the peak at 50 ps as the representative value for an average EET to the CC in C_1_S_1_ from any photon harvested by the peripheral antennas over the entire red region of the action spectrum for photosynthesis (600 to 700 nm). This value is the major experimental conclusion of our present study. Furthermore, we also note that the shoulder at ~5 ps may indicate an additional channel of inter-complex EET process. However, as this timescale is well within the window of intra-complex EET processes, a definite assignment cannot be conclusively made here.

### Structure-based calculations

**Fig. 7.**
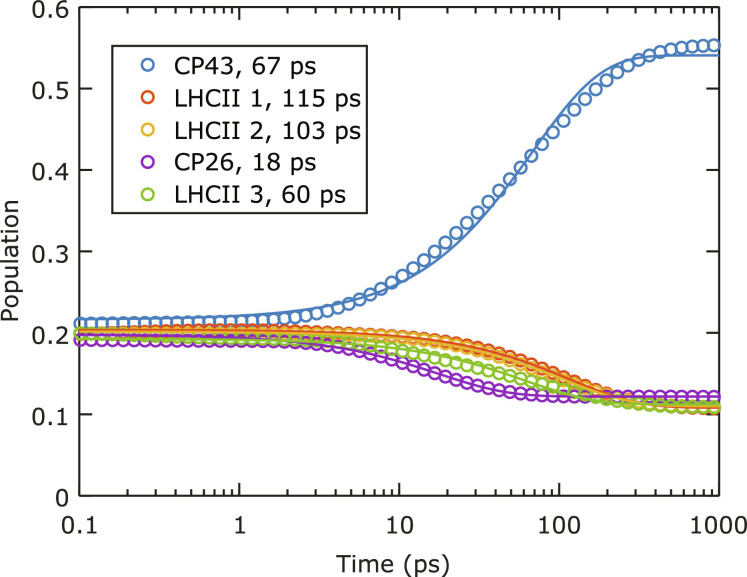
Excited state population kinetics in each pigment-protein complex of C_1_S_1_. The kinetics are calculated using the Redfield–generalized Förster formalism (*59*). The kinetic traces are associated with lifetimes from single-exponential fits. The Chl relaxation rate to ground state is set to zero to better illustrate the inter-complex kinetics.

To obtain a better insight into the EET processes in the C_1_S_1_ complex, we further conduct structure-based calculations. The calculation is based on the Redfield–generalized Förster formalism, often used to describe EET processes in photosynthetic systems (*12*, *52*, *55*, *59*). In particular, Chl pigments are clustered into strongly coupled domains, where EET between excitonic states within a domain is treated with Redfield theory, while EET between the domains is obtained using generalized Förster theory. We perform the calculation on a system consisting of LHCII(S), CP26, and CP43 (chains G-N-Y, S, and C, respectively) from the cryo-EM structure of the PSII supercomplex, with Protein Data Bank code 5XNM (*7*). The parameters used in the calculation, the calculated spectra, and the calculated EET rates are provided in the Supplementary Materials. In Fig. 7, we simulate the disorder-averaged kinetics of the LHCII(S)-CP26-CP43 system and illustrate the excited state population evolution of each monomeric complex. The total population is normalized to unity, and the initial population in each complex is proportional to its total transition dipole strength.

Generally, the populations in all peripheral antenna complexes are partly depleted, while the population in CP43 rises. Here, we use single-exponential fits to find the average population evolution time constants. The fits are applied to data points after *T*_w_ = 1 ps, because inter-complex dynamics largely commences after 1 ps. Among the peripheral antenna complexes, CP26 decays the fastest, with an 18-ps lifetime, evidently due to the close proximity to CP43. Population in LHCII(S) monomer 3, which is the closest to CP43 among the three LHCII monomers (see Fig. 1A), decays in 60 ps, while the other two monomers transfer much slower (>100 ps). The average EET time for the trimeric LHCII(S) is, therefore, around 93 ps. The population of CP43 grows non-exponentially; nevertheless, we obtain a best-fit exponential timescale of 67 ps.

**Fig. 8.**
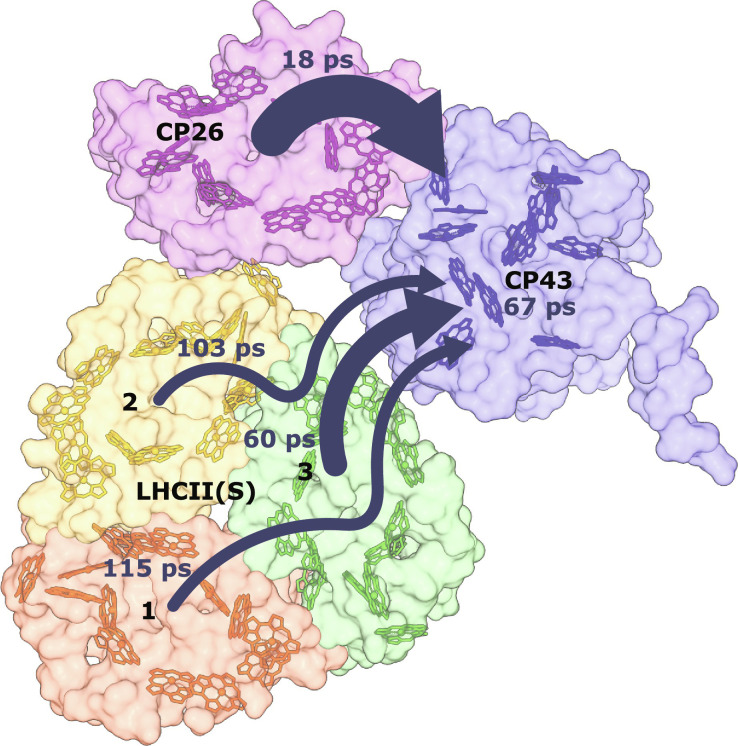
The EET pathways from each monomeric antenna complex to the CC (CP43). Each pathway is associated with a lifetime deduced from the structure-based calculated results. The arrow thicknesses are proportional to the EET rate from each complex.

As shown in fig. S8, the EET dynamics of C_1_S_1_ with individually excited antenna complexes are also simulated. The simulations show that excitations in CP26 are rapidly transferred to CP43, while no population appears in LHCII(S) until much later. In contrast, if either of the LHCII(S) monomers is excited, then equilibration among them occurs fast, followed by EET to CP43 mainly through connections with monomer 3. Therefore, the growth of CP43 population reflects the average of two separate inter-complex EET pathways: from LHCII(S) and CP26 to the CC. Its timescale, 67 ps, can be directly compared with our experimental value of 50 ps, which also represents the average EET time from all antenna complexes. Figure 8 provides the prominence EET pathways taken by each monomeric unit of the C_1_S_1_ complex and their timescales from the calculation.

## DISCUSSION

From previous studies (*10*, *56*), the EET processes within each of the peripheral antenna complexes at 77 K were found to be almost completed within ~10 ps. After *T*_w_ = 10 ps, regardless of the initial excitation conditions, the excitations in the complexes are predominantly in the terminal excitonic state at 678 nm, from which the subsequent inter-complex EET happens. The intra-complex equilibration being faster than the inter-complex EET explains why the effective value of inter-complex EET rate has the same value of ~50 ps over the whole range of excitation wavelengths (Fig. 6A). This observation is consistent with the fact that the terminal Chl domains in the antenna complexes, Chl *a*611-*a*612 and Chl *a*613-*a*614 (*12*, *14*), are at the same time located at the edge of the complex (see fig. S9 for an illustration of the prominent inter-complex Chl connections). These domains, thus, serve as the bridge for EET to the adjacent complexes, and the arrangement facilitates inter-complex EET. It may be illustrative here to consider possible scenarios that may, in principle, lead to the observation of excitation wavelength–dependent dynamics. For instance, consider the scenario where the Chl *a*/*b* ratio of LHCII(S) and CP26 are slightly different, while the transfer rates to the CC are very dissimilar between LHCII(S) and CP26. In such scenario, excitation wavelength–dependent dynamics may be observed. However, our current measurement suggests that the considered scenario may be too simplistic and that the effect is not strong enough to be clearly observable.

For the room-temperature (RT) inter-complex EET timescale, several reports can be found in literature. Using time-resolved fluorescence measurement and a coarse-grained kinetic model, Caffarri *et al.* (*5*) reported a value of 42 ps for the average migration time for an excitation created somewhere in a C_2_S_2_M_2_ supercomplex to reach the RC. The kinetic model assumes that all the inter-complex EET connections are very similar (with small differences arising from the scaling by the number of Chls *a* in the complex). Furthermore, the kinetics fit needed to include parameters related to the charge separation process because the fluorescence decay is also a function of the charge separation process. It is clear, however, from the current structural models that the connections between different complexes are dissimilar. Nonetheless, on the basis of these data (*5*), extrapolation can be made to estimate the effective time it takes for the excitations from the peripheral antennas LHCII(S)-CP26 to transfer to the CC at ~50 ps at RT. Although this is in concurrence with our measured transfer time of 50 ps at 80 K, it is just coincidental, as there are clear differences in our approaches. Our current work aims to measure the transfer rates via more specific inter-complex connections. Here, we were able to measure the averaged transfer rate to the CC from two adjacent complexes LHCII(S) and CP26, thus providing more specific details on inter-complex transfer dynamics than the earlier experiment and model. Our approach does not require any approximation of subsequent processes, such as charge separation.

In a review by Croce and van Amerongen (*6*), the effective migration times from LHCII(S) and CP26 to the CC were mentioned to be 75 and 15 ps, respectively. These values include both the equilibration time within the antenna complex and the inter-complex EET time. The former component can be approximated at 25 ps for trimeric and 10 ps for monomeric complexes (*6*, *60*). From this, one may conclude that inter-complex EET from the LHCII(S) trimer takes 50 ps and that from CP26 takes 5 ps, while the average EET time from both of them is between these values. The RT EET in the PSII supercomplex was also investigated with structure-based and quantum mechanical calculations (*36*, *37*, *39*, *40*, *61*). Modified Redfield–generalized Förster theories were used on PSII C_2_S_2_M_2_ (*36*) to calculate the averaged time for an exciton to diffuse from the peripheral antenna complexes to the CC. The diffusion lifetime was estimated to be around 50 ps when all Chls *a* were equally excited. Similar to the above paragraph, the explicit antenna-to-core EET lifetime can be estimated at around 25 ps by subtracting the 25-ps equilibration time of the LHCII trimer. A HEOM approach was also reported (*39*) to find that excitation from an outer LHCII can populate the inner PSII antenna complexes in 45 ps, and the CC is then populated at 125 ps. The diffusion lifetime from the inner antennas to the CC is thus around 80 ps, giving the antenna-to-core EET timescale to be 55 ps after subtracting the LHCII equilibration time.

Together, previous experimental and theoretical studies yield a timescale range of 25 to 55 ps for the EET from the antenna to the CC at RT. The estimated EET timescale at RT is generally faster compared to our measured EET time of 50 ps at 80 K because, at higher temperatures, the spectral overlap between donor and acceptor states is larger, thus accelerating the resonance energy transfer processes. According to our previous study on the temperature dependence of the EET lifetime in LHCII, the overall EET dynamics were estimated to be around two to three times faster at 295 K than at 77 K (*10*). Thus, although not definite, we can estimate the inter-complex EET lifetime from 50 ps at 80 K to ~17 to 25 ps at physiological conditions. Our structure-based calculations at RT also result in 27 ps for the effective EET time from the antennas to CP43 (see fig. S10).

Given the fact that the average distance from pigments in CP26 to those in CP43 is shorter than that from LHCII(S), it is expected that excited state population transfers faster from CP26 to the CC. Our structure-based calculation indicates a much faster antenna-to-core EET time from CP26 compared to LHCII(S) (18 ps versus 93 ps). In our experimental analysis (Fig. 6C), apart from the major decay component peaking at 50 ps, the lifetime density of α(*T*_w_) exhibits another small contribution in the ~5-ps timescale. This lifetime contribution may be attributed to the fast EET process from CP26 to the CC. However, there is not enough evidence that this decay process is an inter-complex EET process because a concomitant rise in β(*T*_w_) around the same timescale is not clearly observed.

With such strong connections to the CC, CP26 may also serve as an alternative EET pathway for energy from LHCII(S). The connections between LHCII(S) and CP26 can occur between the Chl *a*604 pigments in monomer 2 of LHCII(S) and CP26 (26 Å, Mg-to-Mg distance). The Chl *a*610 pigments in both complexes are also 27 Å apart. However, those distances are large, and, from isolated LHCII studies, Chl *a*604 is expected to have weak intra-complex connections to the other Chl domains (*12*, *14*); hence, it is not well-suited to be the connector to the other protein complexes. In our calculated population dynamics with initial excitation injected selectively in LHCII(S) monomer 2 (see fig. S8), the population can also be transferred to CP26, but the fast equilibration with other LHCII(S) monomers still dominates. Thus, EET between LHCII(S) and CP26 is likely minor, in agreement with (*6*).

The 2DES in our current setup using pulse shaper–assisted pump-probe geometry (*62*, *63*) can be considered a general form of TA spectroscopy. Although selective narrowband TA can be used to measure much of the results of reported here, there are several advantages in using the current 2DES setup (*41*). With one consistent set of measurement, 2DES datasets can simultaneously generate all possible narrowband pump-probe and broad-band pump-probe spectra, facilitating more consistent analysis than between several pump-probe experiments performed over different excitation bandwidth conditions.

In summary, we use 2DES to measure the EET dynamics within the C_1_S_1_ complex and observe energy transfer processes between the peripheral antennas and the CC. The EET process from the LHCII(S)-CP26 antennas to the CC in plant PSII can be directly observed in the 2D spectra, wherein the effective EET timescale between the two is found to be 50 ps at 80 K and is practically independent of the excitation wavelength. Our results show that most inter-complex EET processes happen after the intra-complex energy equilibrations have ceased. Structure-based calculation suggests that this is mainly partitioned between an 18-ps path from CP26 and 93-ps path from LHCII(S) to the CC. We show how our approach of using advanced spectroscopic techniques on various domains of a supercomplex, together with high-level structure-based calculations, allows us to obtain important EET information. The result from this study can be used as a reference for subsequent studies regarding long-range EET in photosynthetic systems. Our method of analyzing the spectral data using the data of its components proves to be effective for large plant photosynthetic complexes, due to inter- and intra-complex EET processes being distinguishable in lifetimes. This will allow simplifying future studies regarding larger systems as the inter-subunit processes may be treated separately and coarse-grained treatments could be applied in theoretical calculations. The approach may also be applied to uncover the EET timescales of other systems constructed with multiple subunits, such as plant photosystem I, phycobilisome in cyanobacteria, the antenna system of purple photosynthetic bacteria (LH1 and LH2), or even artificial systems such as photovoltaic and nanomaterial heterostructures.

## MATERIALS AND METHODS

### 2D electronic spectroscopy

The 2DES setup used a pump-probe beam geometry (*62*, *63*). A Ti:sapphire amplifier (Coherent, Legend) was used to generate 800-nm, 1-kHz pulses. The laser pulses were focused to a 1.2-m pressurized argon tube using a 1-m concave mirror to generate a supercontinuum. The supercontinuum pulse was then split using a wedged glass window, through which most of the pulse is transmitted and used as the pump pulse. The reflected part from the wedged window was used as the probe and reference. The pump pulse was compressed using a single-prism compressor (*64*) and sent to an acousto-optical pulse shaper (Fastlite, Dazzler) to generate a double-pulse train. The double pump pulse train was generated with 1 × 2 phase cycling scheme and an interpulse delay τ varying from 0 to 300 fs with 6-fs steps. The pump pulses after compression and shaping have a bandwidth of ~100 nm (center at 650 nm; Fig. 1B) and duration of ~20 fs as measured with an autocorrelator. The probe pulse was compressed using a pair of dispersion-compensating mirrors (Laser Quantum, DCM10) and retro-reflected on a motorized linear stage (Physik Instrumente) to control the pump-probe delay time *T*_w_. The effective instrument response function, by analyzing the coherent artifact signal from a TA measurement of pure solvent, gives an experimental time resolution of ~44 fs (see fig. S11). The probe was further 50/50 split to create a reference pulse to compensate for the shot-to-shot power fluctuation. The probe polarization was set at magic angle (54.7°) with respect to the pump to eliminate the effects of rotational diffusion during the waiting time. The pump, probe, and reference pulses were focused onto the sample using an off-axis parabolic mirror. The focused pump and probe had radii of 140 and 70 μm, respectively. The pump power was kept below 3 nJ per pulse to reduce sample degradation and exciton-exciton annihilation. In this condition, the average excitation on each protein complex was 5 to 10%. The probe and reference pulses were then recorded on a CCD camera (Princeton Instruments, PIXIS). The pump-probe delay time *T*_w_ was scanned linearly from −100 to 100 fs with steps of 10 fs and then increased exponentially (i.e., linear steps in logarithmic scale) in 56 steps from 100 fs to 1.5 ns. The collected τ-dependent signal Δ*A*(τ, *T*_w_, λ*_t_*) was Fourier-transformed along τ to yield the 2D spectra *S*(λ_τ_, *T*_w_, λ*_t_*). The spectral resolutions were ~2.3 nm (1/2τ_max_) at 650 nm on the λ_τ_ axis and ~0.4 nm on the λ*_t_* axis. TA spectra can be generated with a given excitation (pump) bandwidth from the 2D spectra by integrating *S*(λ_τ_, *T*_w_, λ*_t_*) over the range of given excitation wavelengths λ_τ_.

### Sample preparation

The LHCII trimer, CC, and C_1_S_1_ complexes were prepared with the previously reported protocols used to purify the PSII core and supercomplex with different antenna sizes (*5*, *51*, *65*). For ultrafast 2DES experiments at 80 K, the samples were diluted so that the final solution contains 10 mM Hepes, 0.01% n-dodecyl-α-D-maltopyranoside (α-DDM), and 60% v/v glycerol. The dilution results in *Q_y_* absorption at ~0.13 OD at RT (1-mm optical path). The sample was then squeezed between two sapphire windows with 1-mm Teflon spacer and cooled to 80 K using a liquid nitrogen cryostat (Janis). The 2DES measurement for each complex was repeated at least three times to ensure the reproducibility.
