# Supplementary material for: Inter-subunit energy transfer processes in a minimal plant photosystem II supercomplex
Source: Sci Adv. 2024 Feb 23;10(8):eadh0911. doi: 10.1126/sciadv.adh0911 (PMC10889429; doi:10.1126/sciadv.adh0911)
Supplement: Supplementary file 1 — Supplementary Text Figs. S1 to S11 Tables S1 to S5 Legend for table S6 [file sciadv.adh0911_sm.pdf]

Science Advances
AAAS

# Supplementary Materials for

## Inter-subunit energy transfer processes in a minimal plant photosystem II supercomplex

Hoang Long Nguyen *et al.*

Corresponding author: Howe-Siang Tan, howesiang@ntu.edu.sg

*Sci. Adv.* **10**, eadh0911 (2024)
DOI: 10.1126/sciadv.adh0911

**The PDF file includes:**

Supplementary Text
Figs. S1 to S11
Tables S1 to S5
Legend for table S6

**Other Supplementary Material for this manuscript includes the following:**

Table S6

# Kinetic model for antenna-to-core EET

Considering a two-level system, with two excited states $A$ and $B$, representing the equilibrium states of LHCII and the CC, respectively. Here, each state has its own intrinsic relaxation rate, $k_{isoA}$ and $k_{isoB}$, accounting for the relaxation process in isolated form in each complex. For simplicity, only energy transfer from state $A$ to $B$ is possible. A transfer rate constant, $k_{ICT}$, represents the inter-complex EET rate from LHCII to the CC.

The transfer rate matrix is then:

$$\mathbf{K} = \begin{pmatrix} -k_{isoA} - k_{ICT} & 0 \\ k_{ICT} & -k_{isoB} \end{pmatrix}, \tag{S1}$$

The population evolution in the system is described by the rate equations:

$$\begin{pmatrix} \dot{A} \\ \dot{B} \end{pmatrix} = \mathbf{K} \begin{pmatrix} A \\ B \end{pmatrix}. \tag{S2}$$

whose solutions are:

$$A(t) = A_1 \exp\left[-(k_{ICT} + k_{isoA})t\right], \tag{S3}$$

$$B(t) = B_1 \exp\left[-(k_{ICT} + k_{isoA})t\right] + B_2 \exp\left(-k_{isoB}t\right), \tag{S4}$$

Here, the constants $A_1$, $B_1$ and $B_2$ are defined given the initial populations $A(0)$ and $B(0)$:

$$A_1 = A(0), \tag{S5}$$

$$B_1 = -\frac{k_{ICT}}{k_{ICT} + k_{isoA} - k_{isoB}} A(0), \tag{S6}$$

$$B_2 = B(0) + \frac{k_{ICT}}{k_{ICT} + k_{isoA} - k_{isoB}} A(0). \tag{S7}$$

Note that if we assume $k_{ICT} + k_{isoA} > k_{isoB}$, *i.e.*, the total decay rate of $A$ is faster than $B$, $B_1$ is negative and the first term in $B(t)$ describes a rise in population.

If we express the population kinetics of $A$ as its isolated kinetics scaled with a time-dependent factor $\alpha(t)$, we then get:

$$A(t) = \alpha(t) A_{iso}(t) \tag{S8}$$

$$= \alpha(t) A(0) \exp(-k_{isoA}t). \tag{S9}$$

Therefore, $\alpha(t)$ only contain the evolution of the inter-complex process:

$$\alpha(t) = \exp(-k_{ICT}t). \tag{S10}$$

Similarly, the scaling factor $\beta(t)$ of $B$ can be expressed as:

$$\beta(t) = \frac{B(t)}{B_{iso}(t)} \tag{S11}$$

$$= \frac{B(t)}{B(0)\exp(-k_{isoB}t)} \tag{S12}$$

$$= \frac{B_1}{B(0)}\exp[-(k_{ICT} + k_{isoA} - k_{isoB})t] + \frac{B_2}{B(0)} \tag{S13}$$

$$= -\frac{k_{ICT}}{k_{ICT} + k_{isoA} - k_{isoB}}\frac{A(0)}{B(0)}(1 - \exp[-(k_{ICT} + k_{isoA} - k_{isoB})t]) + 1. \tag{S14}$$

It can be seen that $\beta(t)$ grows with rate $k_{ICT} + k_{isoA} - k_{isoB}$, then stablises at a constant value. The amplitude of the rise is also proportional to the initial population ratio between $A$ and $B$.

# Structure-based calculations

The structure-based calculation uses the Redfield – generalized Förster theories (*14*, *31*, *59*). The protein structure is obtained from the cryo-EM structure of the PSII supercomplex from *Pisum sativum* (PDB code 5XNM). Here, we only consider the reduced group of complexes containing LHCII(S), CP26, and the CC (chain G-N-Y, S, and C, respectively), henceforth called the reduced $C_1S_1$ complex. In this study, the structure-based calculation follows closely the formalism described in reference (*25*), but neglecting the intramolecular vibrations, *i.e.*, the Franck-Condon factors for intramolecular vibrational transitions are set as $FC(0,0) = 1$ and $FC(0,1) = 0$. The coupling cutoff and energy difference cutoff for clustering are respectively 20 and 300 $cm^{-1}$. The protein correlation radius is 0.5 nm. The temperature is 80 K and the pure dephasing time is 0.3 ps. The Chl relaxation rate to ground state is set to 0 to better illustrate the inter-complex kinetics. The square of the transition dipole strength for Chl *a* and *b* are respectively 21 and 15 $Debye^2$. The site energy and standard deviation, as well as Huang-Rhys factor for each Chl pigment is shown in Tables S1 to S5. Parameters for CP43 are taken from reference (*52*). Those for LHCII are taken from reference (*55*) (model A). Those for CP26 are adapted from LHCII, in which the switch from Chl *b* to *a* at site 609 is accounted for by red-shifting the site energy with an amount equal to the difference in average energies of Chl *a* (14900 $cm^{-1}$) and *b* (15385 $cm^{-1}$) (*55*). The Chl pigments are grouped into clusters. EET inside each cluster is treated with Redfield theory, while EET between clusters are treated with generalized Förster theory. The cluster index for each pigment is also shown in Tables S1 to S5. The calculation is repeated 1000 times with randomized site energies following Gaussian distributions with the corresponding centers and standard deviations.

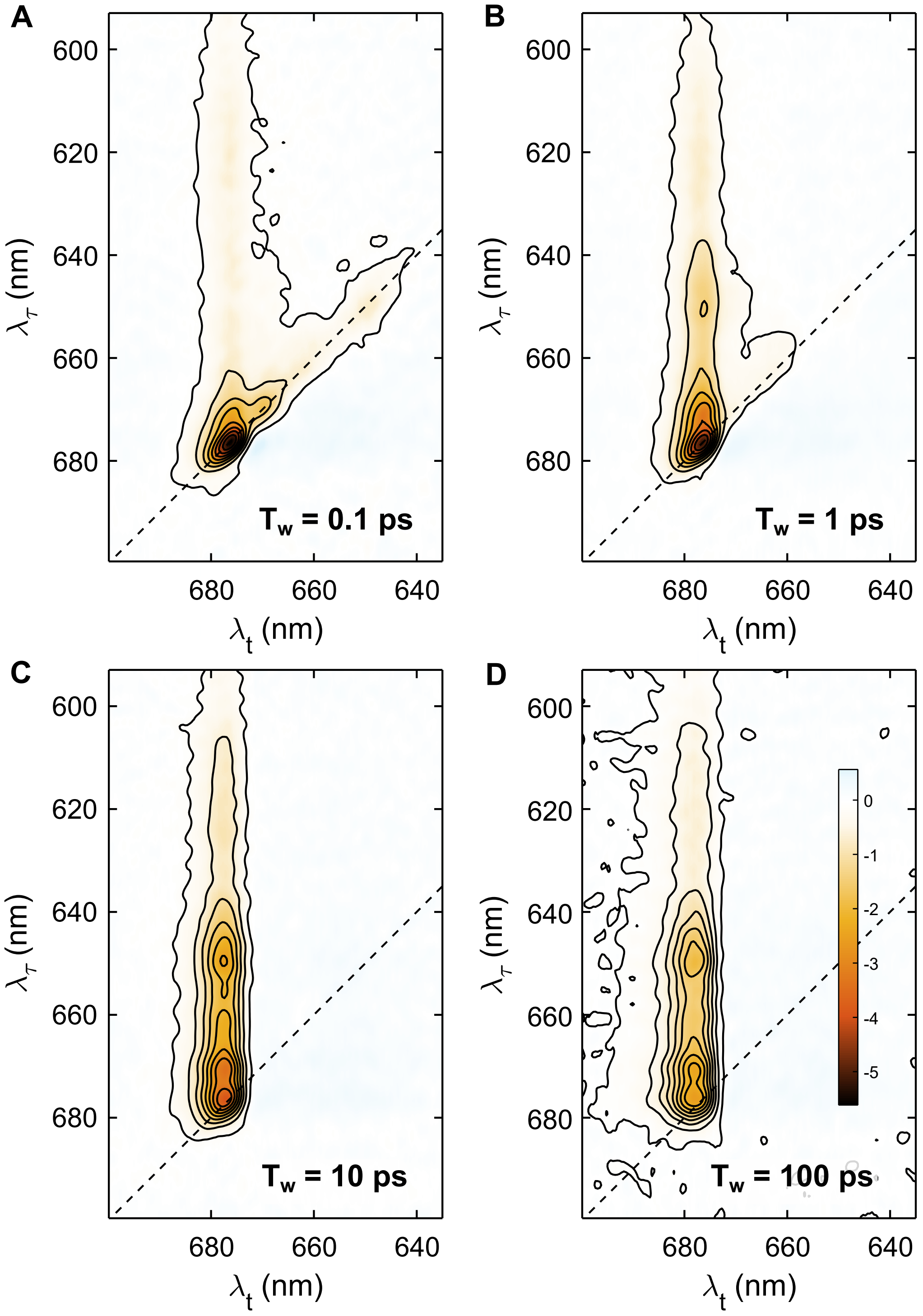


**Figure S1. 2D spectra of LHCII at 80 K.** The spectra are shown at $T_w$ = 0.1, 1, 10, and 100 ps.

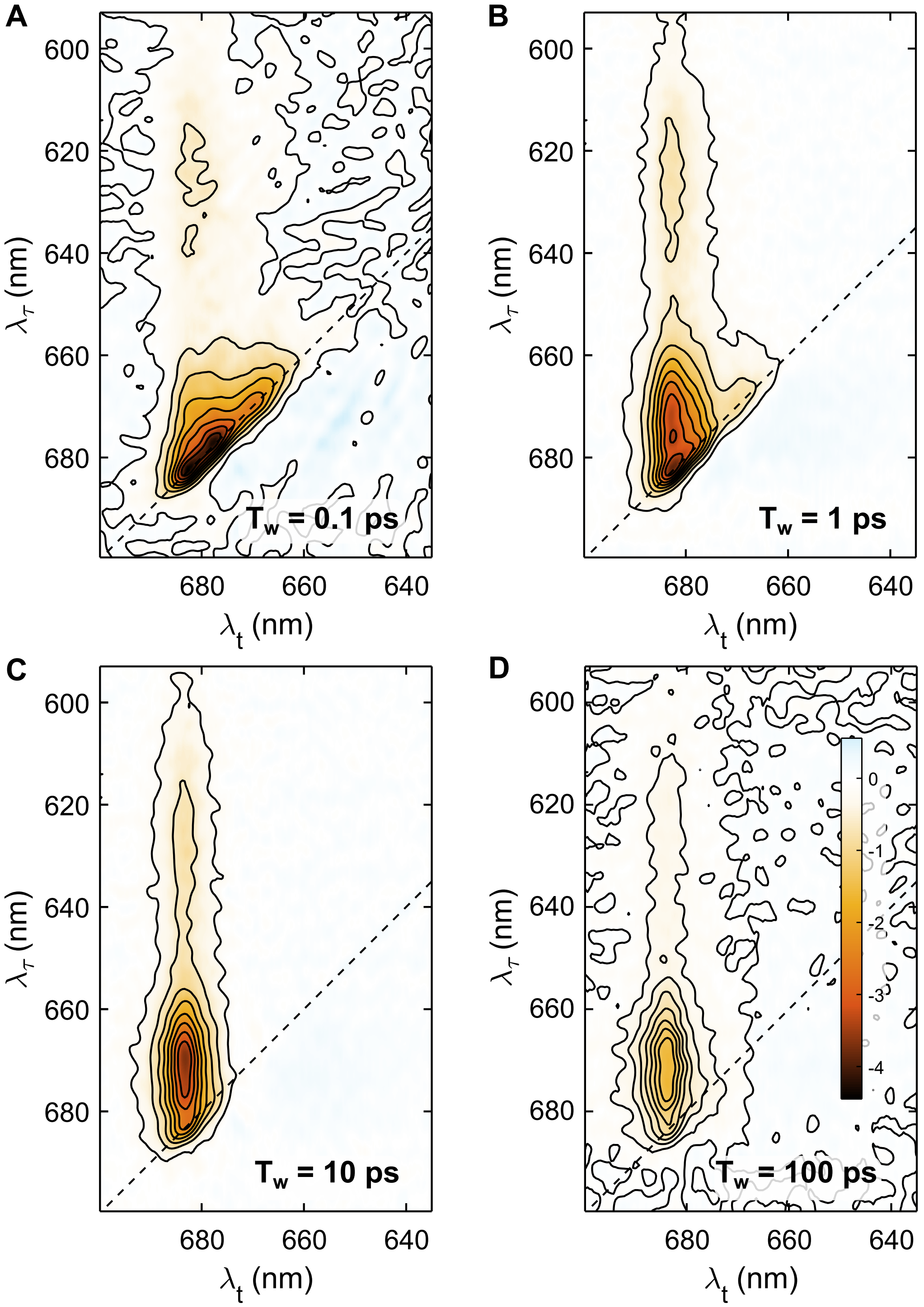


**Figure S2. 2D spectra of the CC at 80 K.** The spectra are shown at $T_w$ = 0.1, 1, 10, and 100 ps.

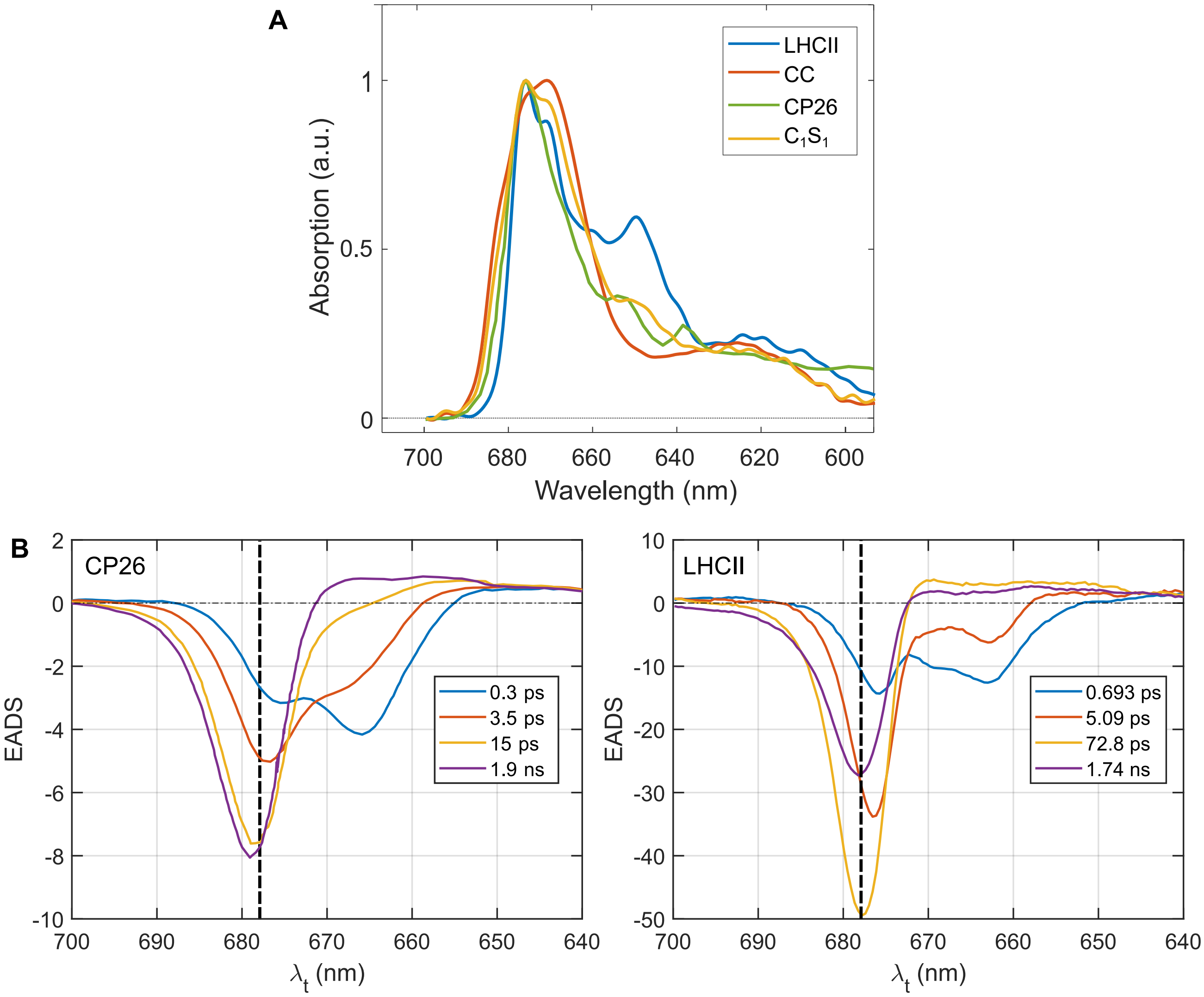


**Figure S3. Comparisons of the spectra of CP26 and LHCII.** (**A**) Linear absorption spectra at 80 K of the LHCII trimer (blue), CC (red), CP26 (green), and $C_1S_1$ complex (yellow), normalized to the $Q_y$ maxima. (**B**) The EADS of CP26 (left) and LHCII (right). The EADS for CP26 are adapted from reference (*20*), with excitation around 661 nm. The EADS for LHCII are taken from the TA spectra of LHCII excited at $\lambda_\tau$ = 656-666 nm. The dashed black line marks the position of $\lambda_t$ = 678 nm.

Figure S3 shows the linear absorption of CP26, obtained from reference (*20*), in comparison with the LHCII, CC, and $C_1S_1$ complex. The evolution-associated difference spectra (EADS) of CP26 (*20*) and LHCII, obtained from the global analysis of their TA spectra, are also compared. They suggest that the spectra and dynamics of CP26, especially after $T_w$ = 10 ps, are reasonably similar to those of LHCII. We note that CP26 exhibits a slight red-shift at late waiting time compared to LHCII.

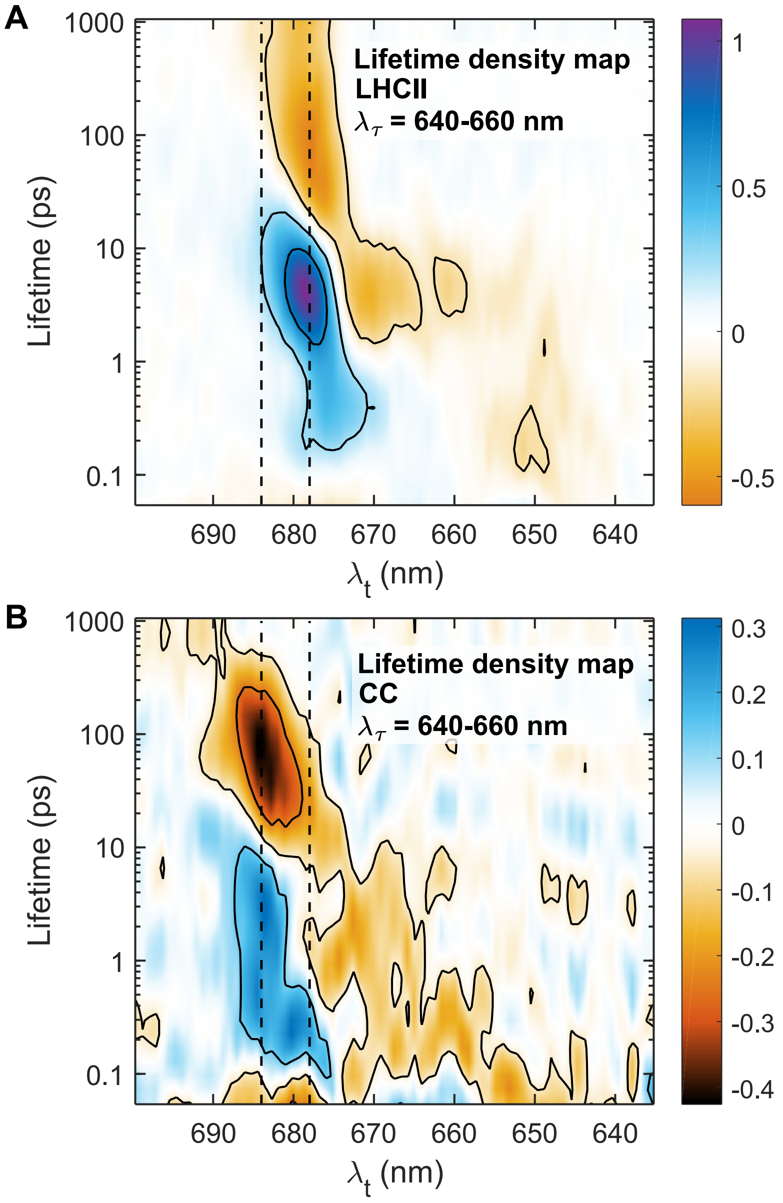


**Figure S4. The LDMs of LHCII and the CC.** The LDMs of (**A**) LHCII and (**B**) the CC with excited energy states $\lambda_\tau$ = 640-660 nm.

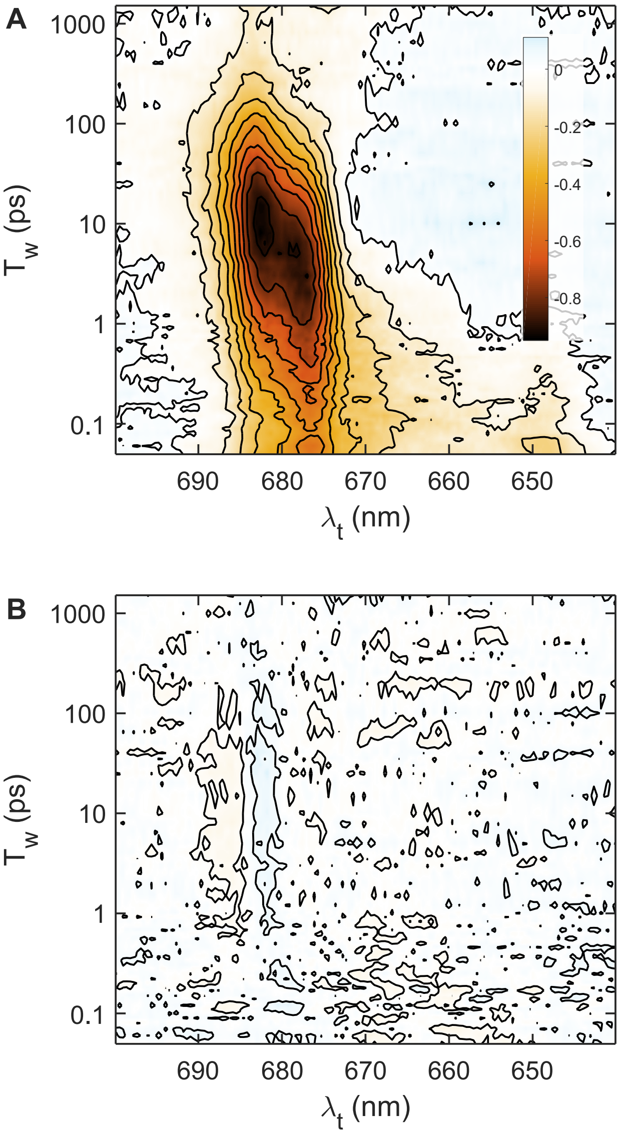


**Figure S5. Quality of the fits of $C_1S_1$ spectra.** (**A**) Experimental time-resolved TA spectra of $C_1S_1$, obtained by integrating the 2D spectra at $\lambda_\tau$ = 640-660 nm. (**B**) The fitting residuals between the fitted and experimental spectra of $C_1S_1$. The color bar is the same as in (**A**) to show the scale of the errors.

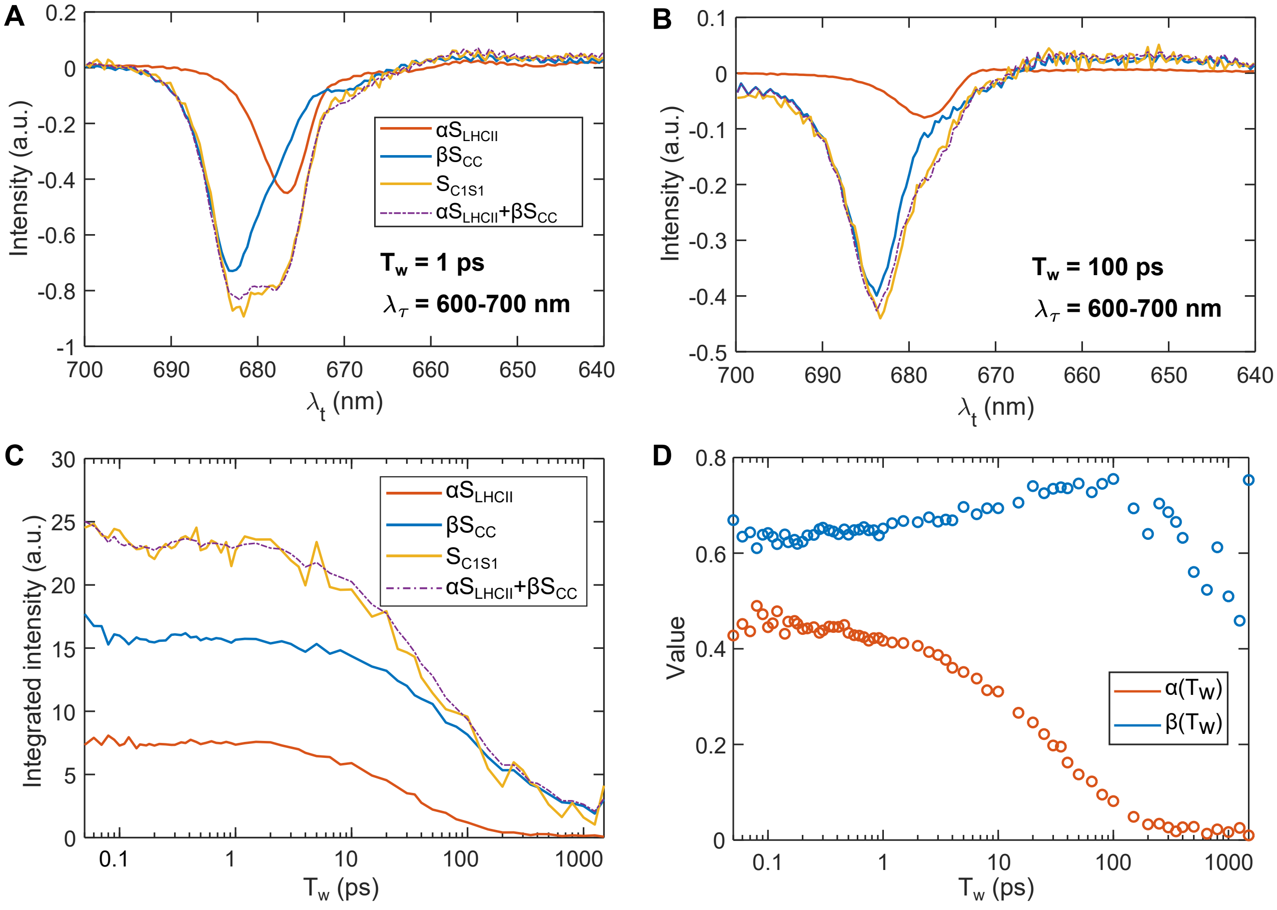


**Figure S6. Results of the fits of $C_1S_1$ spectra with spectra from LHCII and the CC.** (**A**,**B**) TA spectra of $C_1S_1$ at various waiting times $T_w$, each is shown as a linear combination of the spectra from isolated LHCII and CC. The spectra are excited at $\lambda_\tau$ = 600-700 nm. (**C**) Kinetic traces, obtained by integrating the TA signal along $\lambda_t$. (**D**) The values of the scaling factors $\alpha(T_w)$ and $\beta(T_w)$.

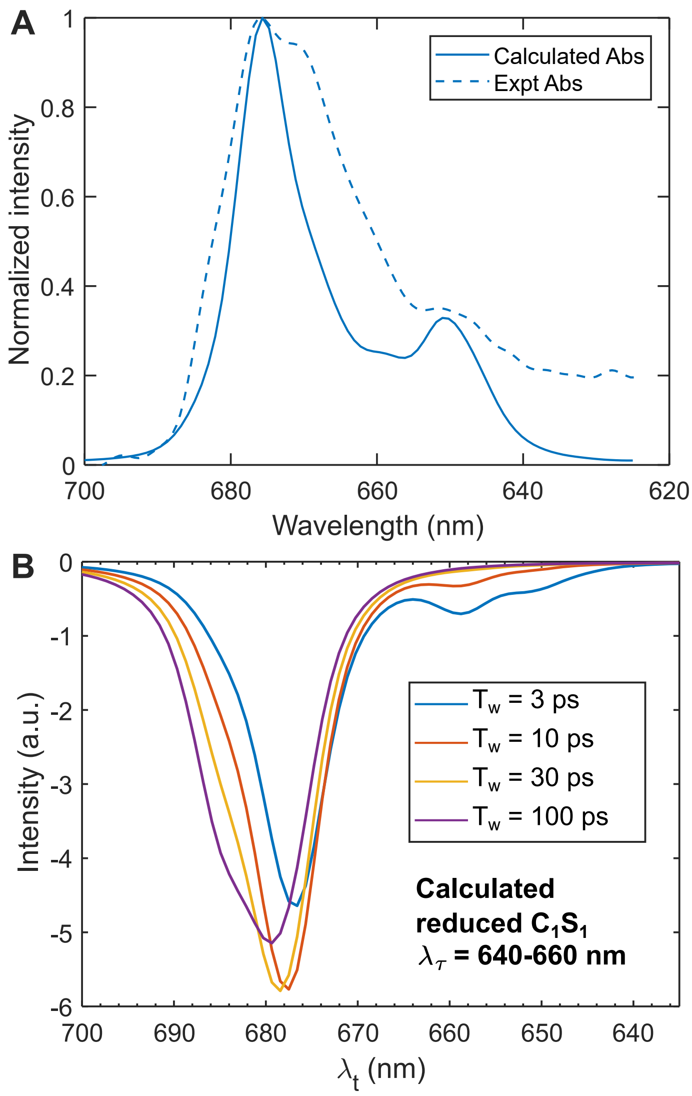


**Figure S7. Calculated spectra from the the structure-based calculation.** Calculated (**A**) linear absorption and (**B**) TA spectra (excited at 640-660 nm) of the reduced $C_1S_1$ complex, obtained from the structure-based calculation.

Figure S7 illustrates the calculated linear absorption and TA spectra of the reduced $C_1S_1$ complex, containing LHCII, CP26, and CP43. The linear absorption is compared with the experimental absorption of the full $C_1S_1$ complex, showing matches at the main Chl *a* and *b* absorption peaks, at 678 and 650 nm, respectively. The calculated absorption peaks are generally narrower than the one from experiment due to the exclusion of CP47 and RC in the calculation, and probably the lack of intravibronic transitions. The calculated TA spectra show concomitant decay and rise of signals at $\lambda_t$ = 678 and 684 nm, respectively, consistent with the experimental spectra.

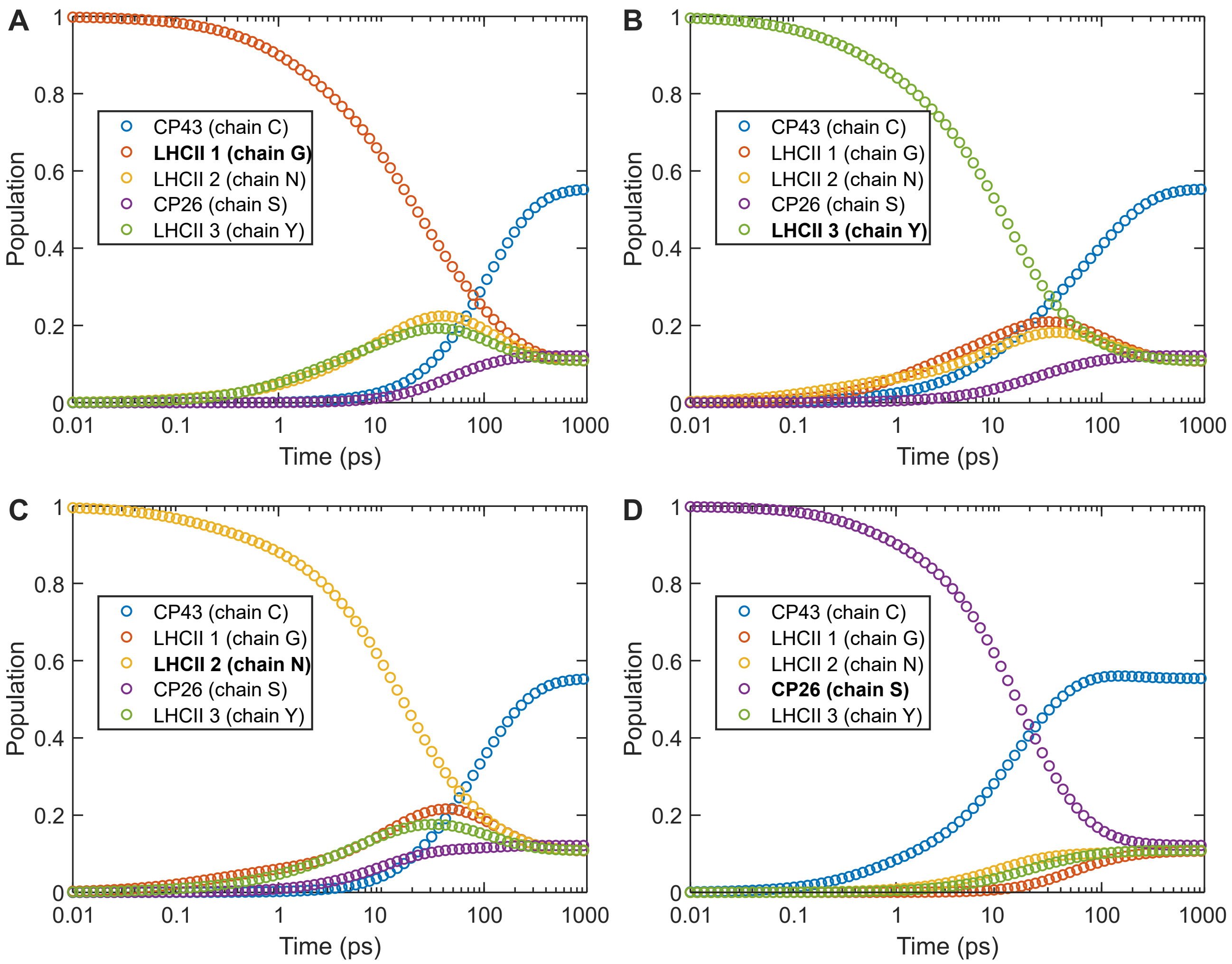


**Figure S8. Calculated population kinetics with initial excitation injected selectively in one monomeric antenna complex**. The excited complex is highlighted in bold font.

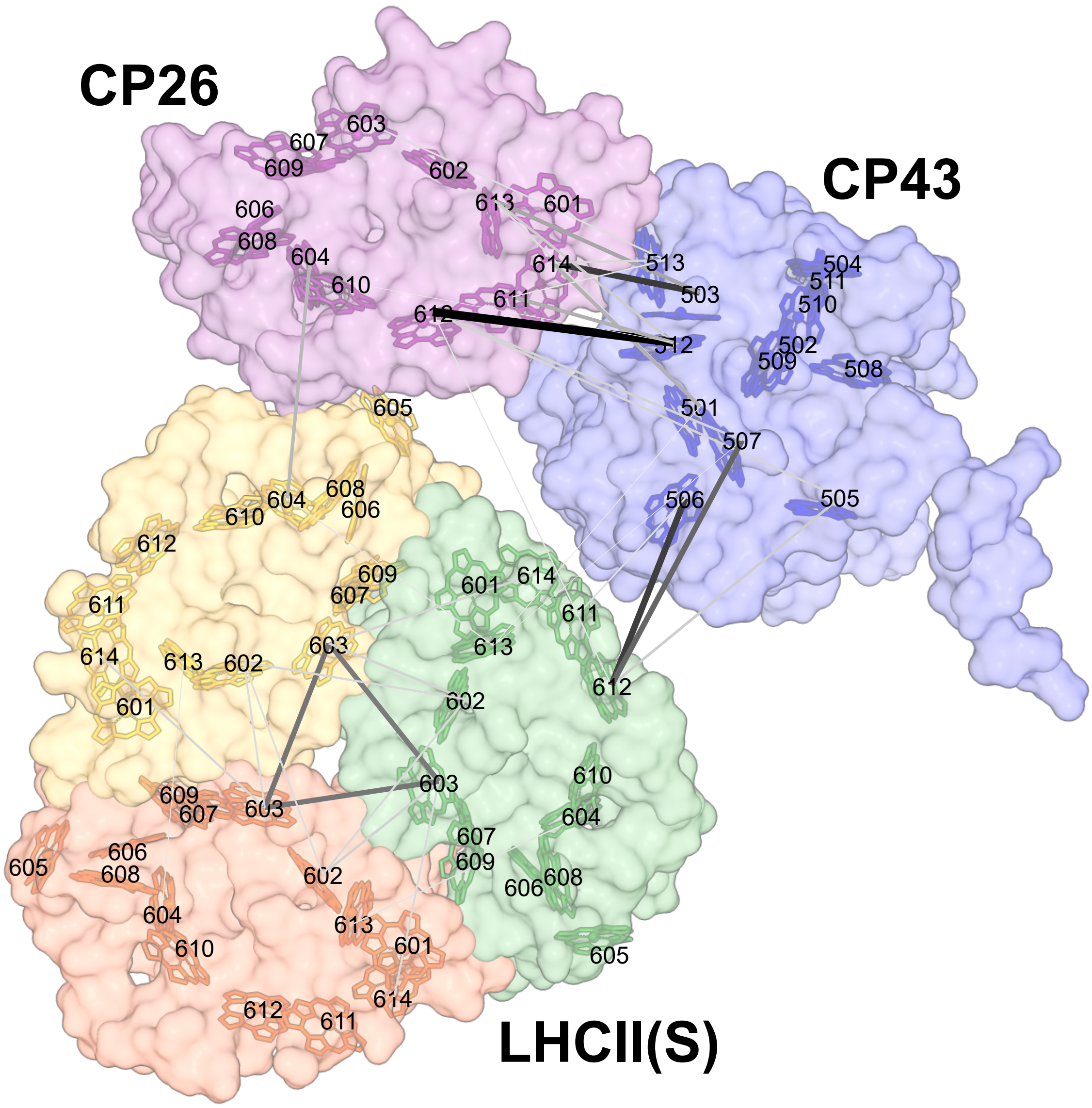


**Figure S9. Illustration of the prominent EET connections between Chls *a* in different complexes of $C_1S_1$.** Faster EET rates are represented by bolder lines.

Figure S9 shows the EET rates between inter-complex Chls *a* pairs, obtained from the structure-based calculation results. The EET rates are the total rates, *i.e.*, sum of forward and backward rate constants. Here, only connections with rate constants faster than $(50\ \mathrm{ps})^{-1}$ are shown, and the fastest rate constant is $(1.6\ \mathrm{ps})^{-1}$ between site 612 in CP26 and site 512 in CP43.

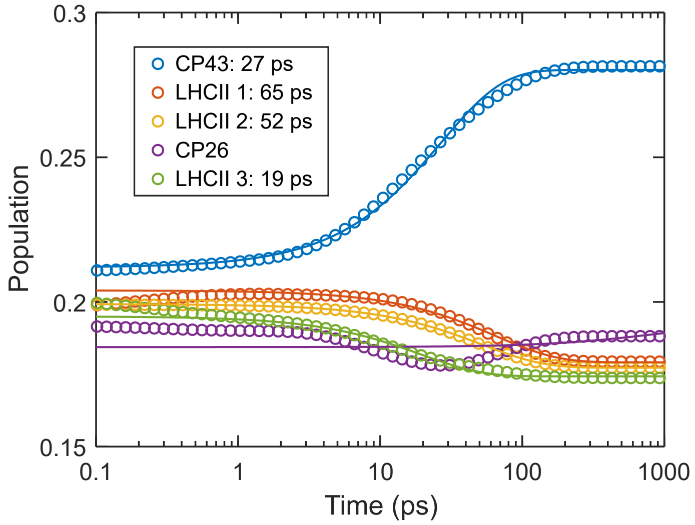


**Figure S10. Calculated population kinetics in each component complex of $C_1S_1$ at room temperature.** The kinetic traces are associated with lifetimes from single-exponent fits.

Figure S10 shows the population kinetics in different protein complexes in the $C_1S_1$ complex, obtained from structure-based calculation at room temperature (with temperature at 300 K and 0.15 ps pure dephasing time). The effective antennae-to-core inter-complex EET is the growth lifetime of CP43 population – 27 ps.

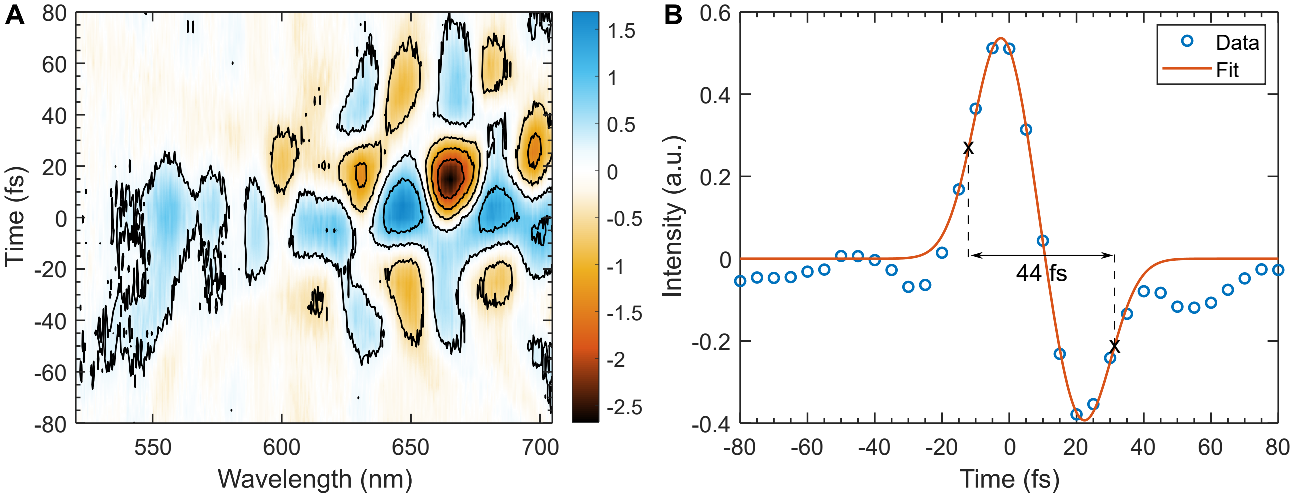


**Figure S11. Coherent artifact signal of a pure solvent.** (**A**) The TA spectra of a pure solvent, showing coherent artifact due to the temporal overlap between the pump and probe pulses. (**B**) The mean coherent artifact signals from all wavelengths, fitted with two Gaussian profiles with the same FWHM.

Figure S11A shows the coherent artifact (CA) signals in a TA measurement of a pure solvent, taken before each collection of 2D data. The CA signals can be considered as a cross-correlation signal between the pump and probe pulses. Panel B shows the integrated signal over the wavelengths, fitted with a pair of Gaussian functions. We can estimate an experimental time resolution of ∼44 fs by taking the difference between the mid-time of the rise in the positive peak and the mid-time of the rise in the negative peak (marked with x's).

**TABLE S1. Site parameters for CP43 (chain C).**

| Chl | Site energy ($cm^{-1}$) | Std dev ($cm^{-1}$) | Wavelength (nm) | Huang-Rhys factor | Cluster number |
|---|---|---|---|---|---|
| 501 | 14859 | 75 | 673 | 0.6 | 23 |
| 502 | 15026 | 75 | 666 | 0.6 | 11 |
| 503 | 15015 | 75 | 666 | 0.6 | 24 |
| 504 | 14749 | 75 | 678 | 0.6 | 11 |
| 505 | 14793 | 75 | 676 | 0.6 | 12 |
| 506 | 14959 | 75 | 669 | 0.6 | 12 |
| 507 | 14848 | 45 | 674 | 0.6 | 12 |
| 508 | 14970 | 75 | 668 | 0.6 | 6 |
| 509 | 14663 | 45 | 682 | 0.6 | 12 |
| 510 | 15337 | 75 | 652 | 0.6 | 25 |
| 511 | 14760 | 75 | 678 | 0.6 | 6 |
| 512 | 14859 | 75 | 673 | 0.6 | 26 |
| 513 | 15083 | 75 | 663 | 0.6 | 27 |

**TABLE S2. Site parameters for LHCII(S) monomer 1 (chain G).**

| Chl | Site energy ($cm^{-1}$) | Std dev ($cm^{-1}$) | Wavelength (nm) | Huang-Rhys factor | Cluster number |
|---|---|---|---|---|---|
| 601 | 15405 | 60 | 649 | 0.5 | 18 |
| 602 | 14940 | 60 | 669 | 0.5 | 20 |
| 603 | 14850 | 60 | 673 | 0.5 | 20 |
| 604 | 14820 | 60 | 675 | 0.5 | 28 |
| 605 | 15465 | 60 | 647 | 0.5 | 15 |
| 606 | 15385 | 60 | 650 | 0.5 | 15 |
| 607 | 15225 | 60 | 657 | 0.5 | 15 |
| 608 | 15215 | 60 | 657 | 0.5 | 19 |
| 609 | 15475 | 60 | 646 | 0.5 | 19 |
| 610 | 14790 | 60 | 676 | 0.5 | 29 |
| 611 | 14950 | 60 | 669 | 0.5 | 4 |
| 612 | 14940 | 60 | 669 | 0.5 | 4 |
| 613 | 14840 | 60 | 674 | 0.5 | 8 |
| 614 | 14940 | 60 | 669 | 0.5 | 8 |

**TABLE S3. Site parameters for LHCII(S) monomer 2 (chain N).**

| Chl | Site energy ($cm^{-1}$) | Std dev ($cm^{-1}$) | Wavelength (nm) | Huang-Rhys factor | Cluster number |
|---|---|---|---|---|---|
| 601 | 15405 | 60 | 649 | 0.5 | 19 |
| 602 | 14940 | 60 | 669 | 0.5 | 21 |
| 603 | 14850 | 60 | 673 | 0.5 | 21 |
| 604 | 14820 | 60 | 675 | 0.5 | 30 |
| 605 | 15465 | 60 | 647 | 0.5 | 13 |
| 606 | 15385 | 60 | 650 | 0.5 | 13 |
| 607 | 15225 | 60 | 657 | 0.5 | 13 |
| 608 | 15215 | 60 | 657 | 0.5 | 17 |
| 609 | 15475 | 60 | 646 | 0.5 | 17 |
| 610 | 14790 | 60 | 676 | 0.5 | 31 |
| 611 | 14950 | 60 | 669 | 0.5 | 1 |
| 612 | 14940 | 60 | 669 | 0.5 | 1 |
| 613 | 14840 | 60 | 674 | 0.5 | 9 |
| 614 | 14940 | 60 | 669 | 0.5 | 9 |

**TABLE S4. Site parameters for CP26 (chain S).**

| Chl | Site energy ($cm^{-1}$) | Std dev ($cm^{-1}$) | Wavelength (nm) | Huang-Rhys factor | Cluster number |
|---|---|---|---|---|---|
| 601 | 15405 | 60 | 649 | 0.5 | 32 |
| 602 | 14940 | 60 | 669 | 0.5 | 5 |
| 603 | 14850 | 60 | 673 | 0.5 | 5 |
| 604 | 14820 | 60 | 675 | 0.5 | 33 |
| 606 | 15385 | 60 | 650 | 0.5 | 16 |
| 607 | 15225 | 60 | 657 | 0.5 | 16 |
| 608 | 15215 | 60 | 657 | 0.5 | 5 |
| 609 | 14990 | 60 | 667 | 0.5 | 5 |
| 610 | 14790 | 60 | 676 | 0.5 | 34 |
| 611 | 14950 | 60 | 669 | 0.5 | 2 |
| 612 | 14940 | 60 | 669 | 0.5 | 2 |
| 613 | 14840 | 60 | 674 | 0.5 | 7 |
| 614 | 14940 | 60 | 669 | 0.5 | 7 |

**TABLE S5. Site parameters for LHCII(S) monomer 3 (chain Y).**

| Chl | Site energy ($cm^{-1}$) | Std dev ($cm^{-1}$) | Wavelength (nm) | Huang-Rhys factor | Cluster number |
|---|---|---|---|---|---|
| 601 | 15405 | 60 | 649 | 0.5 | 17 |
| 602 | 14940 | 60 | 669 | 0.5 | 22 |
| 603 | 14850 | 60 | 673 | 0.5 | 22 |
| 604 | 14820 | 60 | 675 | 0.5 | 35 |
| 605 | 15465 | 60 | 647 | 0.5 | 14 |
| 606 | 15385 | 60 | 650 | 0.5 | 14 |
| 607 | 15225 | 60 | 657 | 0.5 | 14 |
| 608 | 15215 | 60 | 657 | 0.5 | 18 |
| 609 | 15475 | 60 | 646 | 0.5 | 18 |
| 610 | 14790 | 60 | 676 | 0.5 | 36 |
| 611 | 14950 | 60 | 669 | 0.5 | 3 |
| 612 | 14940 | 60 | 669 | 0.5 | 3 |
| 613 | 14840 | 60 | 674 | 0.5 | 10 |
| 614 | 14940 | 60 | 669 | 0.5 | 10 |

**TABLE S6. The calculated, disorder-averaged EET rates between Chl pairs in the the reduced $C_1S_1$ complex, containing LHCII, CP26, and CP43.** The structure-based calculations result in EET rates between exciton pairs, and each exciton is mapped to the most-contributing Chl site. The intra-complex rates are greyed out. The prominent inter-complex rates are highlighted. (Separate file)
